# Supplementary material for: “Energetics of the outer retina II: Calculation of a spatio-temporal energy budget in retinal pigment epithelium and photoreceptor cells based on quantification of cellular processes”
Source: PLoS One. 2025 Jan 27;20(1):e0311169. doi: 10.1371/journal.pone.0311169 (PMC11771881; doi:10.1371/journal.pone.0311169)
Supplement: S1 File — (PDF) [file pone.0311169.s001.pdf]

Supplementary Material

"Energetics of the outer retina II: Calculation of a spatio-temporal energy budget in retinal pigment epithelium and photoreceptor cells based on quantification of cellular processes".

Authors: Christina Kiel, Stella Prins, Alexander JE Foss, Philip J Luthert

Content

Supplementary Text 1

Supplementary Figures S1 to S5

Supplementary text 1: Details of calculations and assumptions taken to calculate the energy budget

(BASAL AND RETINA-SPECIFIC) PLASMA MEMBRANE ION TRANSPORT

Sodium-potassium ATPase: The sodium potassium pump in the PM transports for every ATP 3 Na^+ from inside the cell to out and 2 K^+ from out to inside the cell, which results in a net export of 1 positive charge. The enzyme consists of catalytic subunits (ATP1A1, ATP1A3, ATP1A2, ATP1A4) and non-catalytic subunits (ATP1B1, ATP1B2, ATP1B3, ATP1B4). The maximum rate v_{max} was found to be 2250 (+/- 150) nmol L^{-1} s^{-1}.[1] The k_{cat} can be calculated from $v_{max} = k_{cat} \times [E]$, and with [E] = 50 nM the turnover is 45 s^{-1}.

Calcium ATPases: The plasma membrane calcium ATPase transports for every ATP 1 Ca^{2+} from inside the cell to outside. The transporter binds Ca^{2+} with high affinity, but does not have a high catalytic activity, thereby with low Ca^{2+} concentration keeps Ca^{2+} levels very low inside cells. There are six isoforms (ATP2B1, ATP2B2, ATP2C1, ATP2B4, ATP2B3, ATP2C2). The catalytic activity is between 0.1 and 3 micromol ATP mg $protein^{-1}$ min^{-1} depending on Ca2+ concentration (increasing from 0.1 to 10 micromolar Ca^{2+}).[2] The molecular weight of PM calcium ATPase is 140 kDa.

Dark current (sodium) in rPR cells: Using data from Hagins et al,[3] as quoted by Aidley,[4] one can derive an estimate for the dark current in the rat retina. The maximum longitudinal current is 400 μA/cm^2 and, given a rod density of 10^7/cm^2, this gives a current of 40 pA/rod. 85% of this current is carried by sodium (=34 pA) and 15% is carried by calcium (= 6 pA). The 34 pA of the sodium current are removed by the Na/K-ATPase. The 6 pA of the calcium current are removed by the Ca2+/Na+ exchanger (4 Na+ in for 1 Ca2+ out) and the incoming sodium needs to be removed by the Na/K-ATPase. The 6 pA that are handled by the Na+/K+ ATPase must be adjusted (multiplied by 2) as the charge ratio is 4 to 2 (4 Na+ for 1 Ca2+). This results in 12 pA. The total current that is handled by the Na+/K+ ATPase is therefore 34 pA + 12 pA = 46 pA. There is also some potassium leakage suggesting an underestimate of 7%. Therefore, we need to increase 46 pA by 7%, which is 49.22 pA. The Na/K ATPase pumps out 3 sodium ions per ATP and it is 1 ATP for the calcium and therefore to calculate ATP fluxes, the current needs to be divided by 3 (= 16.41 pA = 16.41 pC s^{-1}). Dividing by Faraday's constant (16.41 pC s^{-1} / 96485 C mol^{-1}) and multiplying by

Avogadro's constant (= 0.00017 pmol s^{-1}) given an ATP consumption rate of 1.02 x 10^8 ATP molecules s^{-1} rod^{-1}.

This is very similar to the estimate of 5.7 × 10^7 ATP molecules/second/rod.[5] The ATP utilisation drops by 2.3 × 10^6 ATP molecules/second/rod per pA decrease and reaches zero at light (10^4 Rh* s^{-1}, where Rh* corresponds to the number of activated rhodopsin molecules per cell).[5]

Based on enzyme abundancies (there are 5.89 x 10^5 molecules of Na/K ATPase expressed in rPR) and k_{cat} of 45/s this results in a flux of 2.65 x 10^7 ATP/s/cell which is 2-fold lower. This is within measurement error. We considered as final value the average between 9.5 × 10^7 ATP molecules/second/rod and 5.7 × 10^7 ATP molecules/second/rod (= 7.6 × 10^7 ATP molecules/second/rod).

<u>Sodium-potassium ATPase (basal = indoor light) in rPR cells</u>: A basal Na/K ATPase activity at daytime (indoor light conditions) was previously estimated to be 5 × 10^6 ATP s^{-1} $cell^{-1}$ and 1.5 × 10^7 ATP s^{-1} $cell^{-1}$,[6,7] which results in an average value of 1 × 10^7 ATP s^{-1} $cell^{-1}$.

<u>Calcium ATPases in rPR cells</u>: The calcium current in rods inner segments is close to 4 pA.[8] The mechanism of calcium extrusion is quite different from that in the outer segment. It is by a Ca-ATPase and is one ATP per calcium extruded.[9-11] This gives an estimate of 2.5 × 10^7 ATP/rod/sec. This is very close to the published values of 2.5 × 10^7 and 3.4 × 10^7 ATP/rod/sec.[5,7] The value of energy requirement by calcium at daytime (indoor light conditions) is 2.0 × 10^6 ATP/rod/s.[7]

The calculations based on calcium ATPase protein abundance and k_{cat} seems to underestimate values, which are in the range of 6.2 × 10^4 to 3.9 × 10^6 ATP/rod/s.

<u>Sodium-potassium ATPase (general) in cPR cells</u>: There are 9.41 × 10^5 molecules of Na/K ATPase expressed in cPR cells. Together with a k_{cat} of 45 s^{-1} this results in 9.41 × 10^5 x 45 = 4.23 × 10^7 ATP s^{-1} $cell^{-1}$. This is close to the 5.0 × 10^7 ATP/s/cell for night.[7] We use the average = 4.62 × 10^7 ATP s^{-1} $cell^{-1}$. The value for day (indoor conditions) is 2.5 × 10^7 ATP/cell/s for day.[7]

<u>Calcium ATPases in cPR cells</u>: Both rods and cone inner segments have an inward calcium current in the dark, through voltage gated channels. The calcium current in cone inner segments is close to 50 pA[7] and doing a similar calculation as for the rod gives an estimate of 1.6 x 10^8 ATP/cone/sec. There is a literature[7] value of 9 × 10^7

ATP/cone/sec in dark conditions and we are using an average value of 1.25×10^8 ATP/cone/s. The day value for the calcium inner segment current in cones form the literature is 9×10^7 ATP/cone/s.[7]

Sodium-potassium ATPase (general) in RPE cells:

A previous model[12,13] gives a figure (based on an integrated model of all ion fluxes in RPE) of the ATP requirement of 1.07×10^8 molecules of ATP/s/cell. The k_{cat} calculation together with enzyme abundance (there are 4.56×10^6 molecules of Na/K ATPase expressed in RPE cell) gives an upper limit of (4.56×10^6 x 45 =) 2.05×10^8 molecules of ATP/s/cell (and one would expect a reserve) and is consistent. We work here with the number of 1.07×10^8 molecules of ATP/s/cell.

Calcium ATPases in RPE: There is no good estimate of energy requirement for cell plasma (not ER) calcium ATPase and we are using enzyme abundance × k_{cat} calculation as upper limits. To calculate the number of ATP molecules needed with high catalytic activity (= 3 micromol ATP/mg protein/min), we first calculated the amount of enzyme (in mg) per cell. The total enzyme concentration is 1.04×10^{-6} mol/L. This was multiplied with 140,000 g/mol to get 0.146 g/L. Using the volume of RPE cell in L (= 2.55×10^{-12} L) we calculated the amount of enzyme per cell (= 3.71×10^{-10} mg/cell). This number was multiplied with 3 min^{-1}, resulting in 1.11×10^{-9} micromol ATP min^{-1} $cell^{-1}$ = 1.86×10^{-11} micromole ATP s^{-1} $cell^{-1}$ = 1.86×10^{-17} mol ATP s^{-1} $cell^{-1}$ = 1.12×10^7 ATP s^{-1} $cell^{-1}$. Similar calculation for low catalytic activity (0.1 micromol ATP /mg protein/min), which results in 3.73×10^5 ATP s^{-1} $cell^{-1}$.

We have a range 3.73×10^5 to 1.12×10^7 ATP/cell/s. We hypothesized that the low value is for dark and the high value is for light due to voltage dependent calcium channels which open with the light rise on EOG.[14]

CYTOSKELETON

Actin fiber polymerization and depolymerization: Actin molecules dissociate from barbed and pointed ends with 1.4/ 0.8/ 7.2/ 0.27 (average = 2.42 s^{-1}) (Bionumbers ID: 107898). The average number of actin filaments was calculated from the lengths (= 4900 nm) and diameter of actin (= 3.5 nm). A filament has two polymers of actin. Hence, an average 4900 nm long actin filaments contains (4900/ 3.5) × 2 = 2800 actin molecules. There are six actin proteins: ACTB, ACTG1, ACTA1, ACTA2, ACTG2,

ACTC1. For the energy budget, we first calculated the number of actin fibers per cell (total number of actin molecules per cell/ 2800). We then assumed that despite 4 ends per actin fiber for polymerization/ depolymerization are available, only 2 ends require 1 ATP hydrolysis. For our energy budget, we assumed basal actin fiber remodeling (using the average dissociation of 2.42 s^{-1}) during the 6 h of night and the first 6 h of day in rPR and cPR cells. In RPE cells, we assumed a similar basal remodeling taking place at night, but because of phagocytosis of outer segments by RPE cells in the morning, we assumed faster actin remodeling in the first 6 h of day (using a dissociation rate of 7 s^{-1}). In the second 6 h of day we assumed fast remodeling (using a dissociation rate of 7 s^{-1}) for all cell types as cytoskeletal remodeling will be important during the anabolic phase where organelle turnover also takes place.

The total number of actin molecules in rPR cells is 1.13×10^6. The number of actin fibers per cell is then 1.13×10^6/ 2800 = 4.02×10^2. Assuming 4 ends per actin fiber for polymerization/ depolymerisation, where only 2 ends require 1 ATP hydrolysis, results in (for night and first 6 h of day) ($4.02 \times 10^2 \times 4 \times 2.42/2$ =) 1.95×10^3 ATP s^{-1} $cell^{-1}$. During the last 6 h of day this results in a budget of ($4.02 \times 10^2 \times 4 \times 7.2/2$ =) 5.79×10^3 ATP s^{-1} $cell^{-1}$.

The total number of actin molecules in cPR is 8.25×10^5. The number of actin fibers per cell is 8.25×10^5/2800 = 2.96×10^2. This results in ($2.96 \times 10^2 \times 4 \times 2.42/2$ =) 1.43 $\times 10^3$ ATP s^{-1} $cell^{-1}$. During the last 6 h of day this results in a budget of ($2.96 \times 10^2 \times 4 \times 7.2/2$ =) 4.26×10^3 ATP s^{-1} $cell^{-1}$.

The total number of actin molecules in RPE is 3.18×10^7. The number of actin fibers per cell is 3.18×10^7/ 2800 = 1.14×10^4. This results in ($1.14 \times 10^4 \times 4 \times 2.42/2$ =) 5.50×10^4 ATP s^{-1} $cell^{-1}$ (at night and the 1st 6 h of day). During the last 6 h of day this results in a budget of ($1.14 \times 10^4 \times 4 \times 7.2/2$ =) 1.64×10^5 ATP s^{-1} $cell^{-1}$.

<u>Transport by myosin molecules on actin filaments</u>: The turnover of Myosin VI is 8.3 s^{-1}.[15] Not all myosins act as monomers. Myosin 1 family members (MYO1A, MYO1H, MYO1G, MYO1F, MYO1C, MYO1B, MYO1D, MYO1E) act as monomer. Myosin 2 family members (MYH1, MYH2, MYH3, MYH4, MYH6, MYH7, MYH7B, MYH8, MYH9, MYH10, MYH11, MYH13, MYH14, MYH15) act as dimer. Myosin 3 family members (MYO3A, MYO3B) are not acting as motor proteins (they are involved in crosslinking of actin) and were not considered here. Myosin 5 family members (MYO5A, MYO5B,

MYO5C) act as dimer. Myosin 6 (MYO6) acts as dimer. Myosin 7 family members (MYO7A, MYO7B) are not well characterized. Myosin 9 family members (MYO9A, MYO9B) act as monomer. Myosin 10 (MYO10) acts as dimer. Myosin 15 (MYO15A, MYO15B) is involved in structural functions (not considered as motor protein here). Myosin 16 (MYO16) was assumed to act as monomer. Myosin 18 (MYO18A, MYO18B) was assumed to act as monomer. Myosin 19 (MYO19) was assumed to act as monomer. Myosin light chains associate with myosins to build functional enzymes.

To calculate the energy budget, the sum of all myosin motors working as monomer were multiplied with k_{cat} of 8.3 s^{-1}. The sum of all myosin motors working as dimers were divided by 2 and multiplied with k_{cat} of 8.3 s^{-1}. This results in a budget of 8.04 × 10^6 ATP s^{-1} $cell^{-1}$ in rPR cells, 3.18 × 10^6 ATP s^{-1} $cell^{-1}$ in cPR cell and 2.82 × 10^7 ATP s^{-1} $cell^{-1}$ in RPE cells.

To check if the numbers are feasible (as they are based on enzyme abundance and k_{cat}), we compared them to the total lengths of actin fibers per cell and the step sizes that myosin motors typically do on actin fibers. Myosins can either do short step sizes of 5 nm or longer step sizes of 36 nm.[16] With a number of 4.02 × 10^2 actin fibers per cell in rPR cells and an average lengths of 4900 nm per fiber, this results in a total lengths of 1.97 × 10^6 nm actin fibers per cell. With a small step size of 5 nm the maximum number of myosin motors that could fit onto this actin fiber network is 3.94 × 10^5, which is 2.5-fold lower than what is available based on the abundance of myosin motor units (9.69 × 10^5) [Note: such a reserve of the cargo would make biological sense]. With a longer step size of 36 nm the number of myosin units that could fit (5.47 × 10^4) is 17.7-fold lower compared to what is available. Similar numbers were obtained for cPR cells, where the number of myosin units that could fit on the actin fiber network is 2.90 × 10^5 (for short step size) and 4.03 × 10^4 (for longer step sizes) compared to a total number of 3.81 × 10^5 myosin motor units, which is 1.3 or 9.5-fold higher than available space. For RPE cells, there is less excess of cargo. The total length of the actin fiber network is 1.14 × 10^4 × 4900 = 5.59 x 10^7 nm per cell. With a small step size this results in a maximum number of 1.12 × 10^7 myosin units that can fit and with a large step size 1.55 × 10^6 myosin units can fit. However, there are only 3.38 × 10^6 myosin motor units available, which lowers the factors to 0.3- and 2.2-fold, respectively.

The energy required for moving myosin motors was corrected in rPR and cPR cells by assuming a limitation by available space of the actin fiber network – albeit using the numbers for a small myosin step size. The final energy budget was calculated based on k_{cat} x maximal number of myosin motor units, which is $8.3 \times 3.94 \times 10^5 = 3.27 \times 10^6$ in rPR cells and $8.3 \times 2.90 \times 10^5 = 2.41 \times 10^6$ in cPR cells.

Tubulin fiber polymerization and depolymerization: There are six tubulin families: alpha, beta, gamma, delta, epsilon. The tubulins are (alpha) TUBA1B, TUBA1A, TUBA4A, TUBA1C, TUBA8, TUBA3C, TUBA3D, TUBA3E, TUBA4B, (beta) TUBAL3, TUBB4B, TUBB3, TUBB2A, TUBB, TUBB2B, TUBB4A, TUBB6, TUBB1, TUBB8, TUBB8B, (gamma) TUBG1, TUBG2, (delta) TUBD1, (epsilon) TUBE1. Members of the gamma tubulin ring complex (no GTPase) are TUBGCP2, TUBGCP3, TUBGCP5, TUBGCP4, TUBGCP6.

For estimating the energy budget, we used an average microtubule length of 4000 nm.[17] The subunit size (dimer) of tubulin is 4 nm (Bionumbers ID: 105534). With 13 subunits (= 26 tubulin molecules) per 4 nm lengths, a tubulin fiber of 4000 nm length contains 1000 × 26 = 26000 tubulin molecules. Microtubules grow at an average rate of 0.3 micrometer per second (and disassemble much faster).[18] Assembly requires GTP. For the energy budget, per cell type we calculated the number of tubulin fibers per cell, which corresponds to the number of tubulin molecules per cell/ 26000. A section of length 4 nm contains 26 tubulin molecules. Growth at speed of 300 nm s^{-1}, results in 300 / 4 (layer of tubulin) = 75 layers with each 26 tubulin s^{-1}. The energy can be calculated from this equation, where f corresponds to the number of tubulin fiber per cell: $f \times 75 \times 26$ GTP s^{-1}.

In rPR the number of tubulin fibers per cell is $3.45 \times 10^6/26000 = 1.33 \times 10^2$ tubulin fibers per cell. This results in a budget of $1.33 \times 10^2 \times 75 \times 26$ GTP $s^{-1} = 2.59 \times 10^5$ GTP = 2.59×10^5 ATP s^{-1} $cell^{-1}$.

In cPR the number of tubulin fibers per cell is $2.10 \times 10^6/26000 = 8.09 \times 10^1$ tubulin fibers per cell. This results in a budget of $8.09 \times 10^1 \times 75 \times 26$ GTP $s^{-1} = 1.58 \times 10^5$ GTP = 1.58×10^5 ATP s^{-1} $cell^{-1}$.

In RPE the number of tubulin fibers per cell is $2.31 \times 10^7/26000 = 8.88 \times 10^2$ tubulin fibers per cell. This results in a budget of $8.88 \times 10^2 \times 75 \times 26$ GTP $s^{-1} = 1.73 \times 10^6$ GTP = 1.73×10^6 ATP s^{-1} $cell^{-1}$.

Transport by kinesin molecules on microtubules: Each kinesin dimer hydrolyses ~ 100 ATP s^{-1}.[6] Another reference suggests microtubule-stimulated k_{cat} values of kinesins between 0.5 and 80 s^{-1}.[19] The following isoforms exist: KIF1B, KIF21A, KIF1A, KIF3A, KIF5B, KIF5C, KIF2A, KIF3C, KIF5A, KIF3B, KIF21B, KIF22, KIF13A, KIF1C, KIF9, KIFC3, KIF16B, KIF26A, KIF6, KIF13B, KIF17, KIF19, KIF20B, KIF27, KIFC2, KIF7, KIF26B, KIF20A, KIF14, KIF4B, KIF18A, KIF25, KIF15, KIF11, KIF2C, KIF24, KIF4A, KIF23, KIFC1, KIF12, KIF18B, KIF2B.

Similar as done for myosin motors, we first wanted to assess the number of kinesin motors present on microtubules. Based on the number of tubulin fibers of 1.33 × 10^2 (rPR), 8.09 × 10^1 (cPR), and 8.88 × 10^2 (RPE), and a 4000 nm length per fiber, this results in total of 5.32 × 10^5 nm (rPR), 3.24 × 10^5 nm (cPR), and 3.55 × 10^6 nm (RPE) total fiber length per cell. Using a step size of 8.1 nm for kinesin motors,[20] this results in a number of kinesin monomers of (total fiber length / 8.1 / 2 =) 1.31 × 10^5 (rPR), 7.99 × 10^4, (cPR), 8.77 × 10^5, (RPE). This is 15.3- (rPR), 32.6- (cPR) and 7- (RPE) fold lower compared to the abundance of all kinesin monomers based on protein abundance.

Microtubules have a relatively big diameter (25 nm) and hence the step size of 4.1 nm might not be a limiting factor for microtubule density when applied only to the length of the microtubule. Therefore, we first calculated the total surface area of microtubules per cell, which is 4.18 × 10^4 µm^2 for rPR cells, 2.54 × 10^4 µm^2 for cPR cells and 2.79 × 10^5 mm^2 for RPE cells. Next we calculated the number of kinesin monomers based on low (5 molecules per µm^2 tubulin area) and high (100 molecules per µm^2 tubulin area) densities.[21] At low density, the number of kinesin molecules is 2.09 × 10^5 (rPR), 1.27 x 10^5 (cPR), and 1.39 × 10^6 (RPE). At high density, the number of kinesin molecules is 4.18 x 10^6 (rPR), 2.54 × 10^6 (cPR), and 2.79 × 10^7 (RPE). These data show that the number of kinesin molecules based on protein abundance are in a similar magnitude compared to those based on high density (see summary in Supplementary Fig. S1).

The assumptions we take for the energy budget is that at night (for all cell types) and during the first 6 h of day (for rod and cone PR cells) there is a low density of kinesin motors and a low k_{cat} (0.5 s^{-1}). This results in a budget of 5.54 × 10^4 ATP s^{-1} $cell^{-1}$ (rPR), 3.37 × 10^4 ATP s^{-1} $cell^{-1}$ (cPR), and 3.70 × 10^5 ATP s^{-1} $cell^{-1}$ (RPE). At day during the second 6 h period (and additionally for RPE cells in the first 6 h period), we assumed

an intermediate number of kinesin molecules on microtubules (indeed, the average between the number of kinesin monomers based on high and low densities is in a similar order of magnitude of < 5-fold as half of the kinesin monomers based on total protein abundance. We therefore assumed that half of the monomers based on protein abundance are moving on microtubules. This assumption is reasonable to still have some kinesin without cargo or with cargo but not on microtubule. We also assumed a higher k_{cat} (average of 0.5 and 80 s^{-1} = 40.25 s^{-1}). This results in a budget of 2.02 × 10^7 ATP s^{-1} $cell^{-1}$ (rPR), 2.62 × 10^7 ATP s^{-1} $cell^{-1}$ (cPR), and 6.13 × 10^7 ATP s^{-1} $cell^{-1}$ (RPE).

<u>Transport by dynein molecules on microtubules</u>: Dyneins are classified into two groups, cytoplasmic and axonemal (or ciliary/ flagellar) dyneins. Only the heavy chains contain the ATPase activity. The following cytoplasmic heavy chain dyneins exist: DYNC1H1, DYNC2H1. The intermediate chains are: DYNC1I1, DYNC1I2. The light intermediate chains are: DYNC1LI1, DYNC1LI2, DYNC2LI1. The light chains are: DYNLL1, DYNLL2, DYNLRB1, DYNLRB2, DYNLT1, DYNLT3. The following axonemal heavy chains exist: DNAH1, DNAH2, DNAH3, DNAH5, DNAH6, DNAH7, DNAH8, DNAH9, DNAH10, DNAH11, DNAH12, DNAH14, DNAH17. The following intermediate chains exist: DNAI1, DNAI2. The following light intermediate chain exists: DNALI1. The following light chains exist: DNAL1, DNAL4. The motor activity is between 0.75 and 15.18 (=8) ATP $motor^{-1}$ s^{-1} depending on concentration of microtubules.[22]

The total number of heavy chain dynein molecules is 2.40 x 10^5 (rPR), 2.00 x 10^5 (cPR), and 4.84 × 10^5 (RPE). This is ~ 10-fold below the concentration of total kinesin monomers and therefore there should be space enough for dynein motors to bind at the same time as kinesin motors. Similar as for kinesin motors, we assumed at the night and the first 6 h of day (for rod and cone PR cells) to have a low density of dynein motors (we assume 10% of the total number of dynein heavy chains as for kinesins at low density approximately 10% of total kinesin monomers are located on a microtubule surface). For the energy budget, this translates into 2.40 × 10^5 / 10 × 8 = 1.92 x 10^5 ATP s^{-1} $cell^{-1}$ (rPR), 2.00 × 10^5 / 10 × 8 = 1.60 x 10^5 ATP s^{-1} $cell^{-1}$ (cPR), and 4.84 × 10^5 / 10 × 8 = 3.87 × 10^5 ATP s^{-1} $cell^{-1}$ (RPE). For the second 6 h of the day cycle (and additionally the first 6 h of day for RPE cells similar as for kinesin motors), we assume that half of the dynein molecules are present on microtubules. This results in a budget

of 2.40 × 10^5 / 2 × 8 = 9.60 × 10^5 ATP s^{-1} $cell^{-1}$ (rPR), 2.00 × 10^5 / 2 × 8 = 8.00 x 10^5 ATP s^{-1} $cell^{-1}$ (cPR), and 4.84 × 10^5 / 2 × 8 = 1.94 × 10^6 ATP s^{-1} $cell^{-1}$ (RPE).

Membrane trafficking accounting for Rab and Arf GTPases: There are 29 Arf GTPases (ARL3, ARL4D, ARF1, ARL2, ARL13B, ARL8B, ARF4, ARL1, ARL6, SAR1A, SAR1B, ARF3, ARL8A, ARL5A, ARF6, ARF5, ARL4A, ARL5B, ARL15, ARL16, ARL4C, ARL17A, ARL17B, ARL9, ARL10, ARL11, ARL5C, ARL13A, ARL14) and 68 Rab GTPases (RAB18, RAB1A, RAB2A, RAB6A, RAB7A, RAB6B, RAB11A, RAB10, RAB14, RAB5A, RABL3, RAB31, RAB4A, RAB5C, RAB21, RAB5B, RAB11B, RAB27A, RAB1B, RAB34, RAB29, RAB13, RAB32, RAB8B, RAB3C, RAB8A, RAB22A, RAB35, RAB9A, RAB33B, RABL6, RAB40B, RAB2B, RAB12, RAB4B, RAB30, RAB28, RAB27B, RAB38, RAB39B, RAB24, RAB3D, RABL2B, RAB23, RAB40C, RAB37, RAB20, RAB15, RABL2A, RAB36, RAB42, RAB9B, RAB17, RAB33A, RAB3B, RAB25, RAB3A, RAB26, RAB41, RAB39A, RAB40AL, RAB6C, RAB6D, RAB19, RAB40A, RAB43, RAB44, RAB7B). GTPase are predicted to cycle efficiently within protein complexes with an average rate of 0.3125 s^{-1}.[23] To calculate the energy budget the different Rab and Arf GTPases were summed up and multiplied with 0.3125 s^{-1}. We assumed that during the night (for all cell types) and during the 1st 6 h of day period (for rod and cone PR cells) only half of the Arf and Rab proteins are cycling. The sum of Rab and Arf GTPases are 7.74 × 10^6 (rPR), 6.74 × 10^6 (cPR), and 2.68 × 10^7 (RPE). This results in a budget of (7.74 × 10^6 / 2 × 0.3125 s^{-1}) = 1.21 × 10^6 ATP s^{-1} cell-1 for rPR cells, of (6.74 × 10^6 / 2 × 0.3125 s^{-1}) = 1.05 × 10^6 ATP s^{-1} $cell^{-1}$ for cPR cells, and of (2.68 × 10^7 /2 × 0.3125 s^{-1}) = 4.20 × 10^6 ATP s^{-1} $cell^{-1}$ for RPE cells.

For the 1st 6 h of day period in RPE cells and for the 2nd 6 h day period (in all cell types), we assumed that all Arf and Rab GTPases are cycling. This results in a budget of (7.74 × 10^6 × 0.3125 s^{-1}) = 2.42 × 10^6 ATP s^{-1} cell-1 for rPR cells, of (6.74 × 10^6 × 0.3125 s^{-1}) = 2.10 × 10^6 ATP s^{-1} $cell^{-1}$ for cPR cells, and of (2.68 × 10^7 × 0.3125 s^{-1}) = 8.39 × 10^6 ATP s^{-1} $cell^{-1}$ for RPE cells.

Rho GTPases: Classical RHO GTPases (RAC1, RAC2, RAC3, RHOA, RHOB, RHOC, CDC42, RHOJ, RHOQ) have intact enzymatic activities and can cycle between GTP-bound and GDP-bound conformations, where an average cycling of 0.3125 s^{-1} was assumed.[23] The sum of classical GTPases is 4.83 × 10^5 (rPR), 3.89 × 10^5 (cPR), and 1.14 × 10^7 (RPE). Fast-cycling Rho GTPases (RHOD, RHOF, RHOU, RHOV) can

freely cycle between GTP-bound and GDP-bound conformations without the involvement of RHOGEFs. An average cycling of 1 s^{-1} was assumed (estimated). The sum of fast cycling Rho GTPases is 2.56×10^4 (rPR), 2.54×10^4 (cPR), and 6.03×10^5 (RPE). We assumed that during the night (for all cell types) and during the 1st 6 h of day period (for rod and cone PR cells) only half of the Rho GTPases are cycling. This results in a budget of ($4.83 \times 10^5 / 2 \times 0.3125 + 2.56 \times 10^4 /2 \times 1\ s^{-1}$) = 8.83×10^4 ATP s-1 cell-1 for rPR cells, of ($3.89 \times 10^5 / 2 * 0.3125 + 2.54 \times 10^4/2 \times 1\ s^{-1}$) = 7.35×10^4 ATP s^{-1} $cell^{-1}$ for cPR cells, and of ($1.14 \times 10^7 / 2 \times 0.3125 + 6.03 \times 10^5 /2 \times 1\ s^{-1}$) = 2.09×10^6 ATP s^{-1} $cell^{-1}$ for RPE cells. For the 1st 6 h of day period in RPE cells and for the 2nd 6 h day period (in all cell types), we assumed that all Rho GTPases are cycling. This results in a budget of ($4.83 \times 10^5 \times 0.3125 + 2.56$ x $10^4 \times 1\ s^{-1}$) = 1.77×10^5 ATP s^{-1} $cell^{-1}$ for rPR cells, of ($3.89 \times 10^5 \times 0.3125 + 2.54 \times 10^4$ x $1\ s^{-1}$) = 1.47×10^5 ATP s^{-1} $cell^{-1}$ for cPR cells, and of ($1.14 \times 10^7 \times 0.3125 + 6.03 \times 10^5 \times 1\ s^{-1}$) = 4.18×10^6 ATP s^{-1} $cell^{-1}$ for RPE cells.

PROTEIN TURNOVER

The energy budget was estimated by taking all ATP (or ATP equivalent) steps into consideration that are required to re-synthesize mRNA, ribosomes, and proteins/ protein folding. Likewise, energy is associated to protein degradation (either via the proteasome or the lysosome) was considered. Degradation of mRNA and ribosomes does not require energy (degradation of ribosomal proteins is already included in the protein degradation budget).

Basic numbers in protein turnover: Protein turnover starts on the level of primary (messenger) RNA transcript production. While the building blocks of DNA are the deoxyribunucleosides (dNTPs) dATP, dGTP, dGTP, dTTP, the building blocks of RNA are the ribonucleosides (NTPs) ATP, GTP, CTP, UTP. Polymerisation of building blocks to either DNA or RNA releases PPi (pyrophosphate). The conversion of NTPs into dNTPs can only happen in the diphosphate form, with NTP -> NDP -> dNTP (by ribonucleotide reductase). On average 2×10^5 mRNA transcripts are in a mammalian cell (Bionumbers ID: 109916). This number is slightly below the value we obtained from the cell biomass composition (Supplementary Table S1), which is 0.53×10^6 molecules for rPR/cPR and 3.64×10^6 molecules for RPE. The ratio of protein to

(primary/m) RNA transcripts is 900.[24] Using this ratio for a number of protein molecules in rPR/cPR of 1.35×10^9 and in RPE of 8.96×10^9, this results in a comparable number of (primary/m) RNA transcripts of 1.50×10^6 (for rPR/cPR) and 9.96×10^6 (for RPE).

To calculate the energy required for mRNA and protein turnover based on demand, we assumed an average lengths of 375 amino (aa) acids per protein.[25] This corresponds to an average length of 3 × 375 = 1125 NTPs for one mRNA transcripts. With an average of 40% of introns,[26] this results in an average length of 1575 NTPs for the primary RNA transcript. mRNA turnover was estimated to be 9 h for all cell types.[23] To estimate protein half-life, for RPE cells we assume 3 days (= 72 h).[27] As 1/10 of outer segments are phagocytosed every day,[28] we assumed a turnover of 10 days (240 h) for the proteins in rPR outer segments. For neurons protein half-life in culture is 5.4 days and 9 days *in vivo*.[29] As total brain includes myelin which contains proteins of very long half-life, used a protein half-life of 5.4 days (129.6 h) for the rest of the rPR and for cPR. As the volume of the outer segment is 91.11 µm3 and the volume of the rest is 403-91.11 = 312 μm^3, 22.6% of total proteins (=3.05×10^8) are in the outer segments and 77.4% (= 1.04×10^9) in the rest of the rPR. There are 1.35×10^9 proteins in cPR. This results in a protein replacement rate of 3.53×10^2 s^{-1} (rPR outer segments), 1.11×10^3 s^{-1} (rest of the rPR), 1.45×10^3 s^{-1} (cPR), and 1.73×10^4 s^{-1} (RPE).

Ribosomes turnover with a half-life of 5 days in fasted rat liver.[30] It is circadian with peak production in the middle of the night for mice which are nocturnal creatures.[31] Other studies measured a half-life of 6 days for brain and 4 days for liver.[32] Here, we assumed a half-life of 6 days for rPR/cPR cells and of 4.5 days for RPE cells.

<u>Messenger (m)RNA turnover – primary transcript production</u>: DNA Helicase is needed for RNA polymerase 2 to expose nucleotides as template. There are 27 DNA helicases in humans[33] (RECQL, BLM, WRN, RECQL4, RECQL5, XRCC6, MCM2, MCM3, MCM4, MCM5, MCM6, MCM7, MCM8, MCM9, MCM10, NCL, CHD2, CHD7, ERCC3, ERCC2, HELLS, INO80, RUVBL1, PIF1, TWNK, BACH1, RTEL1). DNA Helicase hydrolyzes one ATP per nucleotide.[34] For polymerization, NTPs are added, and pyrophosphate is released. Therefore, NMPs are released with mRNA degradation that require each 2 ATP for regeneration of NTPs. This results in a budget of 1 (helicase) + 2 (NMP recycling) = 3 ATP per NTP addition.

Based on a turnover of 9 h and a transcript abundance of 1.50 x 10^6 in rPR cells, this results in a demand of 7.29 x 10^4 NTP s^{-1} $cell^{-1}$. [Note: This is roughly 150-times below the capacity of RNA polymerase 2, which transcribes RNA precursors for mRNA and most sRNA and microRNAs (POLR2E, POLR2K, POLR2G, POLR2L, POLR2B, POLR2J, POLR2A, POLR2F, POLR2H, POLR2C, POLR2M, POLR2J3, POLR2D, POLR2J2, POLR2I). The number of RNA polymerase 2 in rPR is 1.76 × 10^5 and k_{cat} is 60 NTP s^{-1} (http://book.bionumbers.org/what-is-faster-transcription-or-translation/), which results in a theoretically possible rate of transcription of 1.06 × 10^7 NTP s^{-1} $cell^{-1}$.] Based on a turnover demand of 7.29 × 10^4 NTP s^{-1} $cell^{-1}$, this results in a budget of 1 (helicase) + 2 (NMP recycling) = 3 ATP per NTP addition, and a total energy budget of 2.19 × 10^5 ATP s^{-1} $cell^{-1}$. This budget would be for the full 24 hours. However, as the rate of transcription can be 150-times higher, we assumed that transcription happens mainly in the anabolic period (2nd 6 h of day). This results in a budget of 4 × 2.19 × 10^5 = of 8.76 × 10^5 ATP s^{-1} $cell^{-1}$ in 6 h.

Based on a turnover of 9 h and a transcript abundance of 1.50 × 10^6 in cPR cells, this results in a demand of 7.29 × 10^4 NTP s^{-1} $cell^{-1}$. [Note: This is roughly 150-times below the capacity of RNA polymerase 2, which transcribes RNA precursors for mRNA and most sRNA and microRNAs (POLR2E, POLR2K, POLR2G, POLR2L, POLR2B, POLR2J, POLR2A, POLR2F, POLR2H, POLR2C, POLR2M, POLR2J3, POLR2D, POLR2J2, POLR2I). The number of RNA polymerase 2 in cPR is 1.81 × 10^5 and k_{cat} is 60 NTP s^{-1} (http://book.bionumbers.org/what-is-faster-transcription-or-translation/), which results in a theoretically possible rate of transcription of 1.09 × 10^7 NTP s^{-1} $cell^{-1}$.] Based on turnover demand of 7.29 × 10^4 NTP s^{-1} $cell^{-1}$, this results in a budget of 1 (helicase) + 2 (NMP recycling) = 3 ATP per NTP addition, and a total energy budget of 2.19 × 10^5 ATP s^{-1} $cell^{-1}$. Similar as above, we assumed that this step happens mainly in the 2nd 6 h of day, which result in a budget of 4 × 2.19 × 10^5 = of 8.76 × 10^5 ATP s^{-1} $cell^{-1}$ in 6 h.

Based on a turnover of 9 h and a transcript abundance of 9.96 × 10^6 in RPE cells, this results in a demand of 4.84 × 10^5 NTP s^{-1} $cell^{-1}$. [Note: This is roughly 100-times below the capacity of RNA polymerase 2, which transcribes RNA precursors for mRNA and most sRNA and microRNAs (POLR2E, POLR2K, POLR2G, POLR2L, POLR2B, POLR2J, POLR2A, POLR2F, POLR2H, POLR2C, POLR2M, POLR2J3, POLR2D, POLR2J2, POLR2I). The number of RNA polymerase 2 in RPE is 8.04 × 10^5 and k_{cat}

is 60 NTP s^{-1} (http://book.bionumbers.org/what-is-faster-transcription-or-translation/), which results in a theoretically possible rate of transcription of 4.83 × 10^7 NTP s^{-1} $cell^{-1}$.] Based on a turnover demand of 4.84 × 10^5 NTP s^{-1} $cell^{-1}$, this results in a budget of 1 (helicase) + 2 (NMP recycling) = 3 ATP per NTP addition, and a total energy budget of 1.45 × 10^6 ATP s^{-1} $cell^{-1}$. Similar as above, we assumed that this step happens mainly in the 2nd 6 h of day, which result in a budget of 4 × 1.45 x 10^6 = of 5.80 × 10^6 ATP s^{-1} $cell^{-1}$ in 6 h.

Messenger (m)RNA turnover – splicing and nuclear export: The spliceosome requires 12 ATP per intron spiced; such as helicases.[35] Energy from helicase is used for transport out of nucleus (in contrast to other RNAs that require RanGTP cycle). The average number of introns per primary RNA transcript is 8.[36]

Based on a turnover of 9 h and a transcript abundance of 1.50 × 10^6 in rPR cells (= demand of 4.63 × 10^1 transcripts s^{-1} $cell^{-1}$), this results in an energy requirement of 4.63 × 10^1 s^{-1} $cell^{-1}$ × 12 × 8 = 4.44 × 10^3 ATP s^{-1} $cell^{-1}$ in 24 h and in 4 × 4.44 × 10^3 = of 1.78 × 10^4 ATP s^{-1} $cell^{-1}$ in 6 h.

With a number of 1.50 × 10^6 primary transcripts in 9 h in cPR (= demand of 4.63 × 10^1 transcripts s^{-1} $cell^{-1}$), this results in a budget of 4.63 × 10^1 s^{-1} $cell^{-1}$ × 12 × 8 = 4.44 × 10^3 ATP s^{-1} $cell^{-1}$ in 24 h and in 4 × 4.44 x 10^3 = of 1.78 × 10^4 ATP s^{-1} $cell^{-1}$ in 6 h.

With a number of 9.96 × 10^6 primary transcripts in 9 h in RPE cells (=demand of 3.07 × 10^2 transcripts s^{-1} $cell^{-1}$), this results in a budget of 3.07 × 10^2 s^{-1} $cell^{-1}$ × 12 × 8 = 2.95 × 10^4 ATP s^{-1} $cell^{-1}$ in 24 h and in 4 × 2.95 × 10^4 = of 1.18 × 10^5 ATP s^{-1} $cell^{-1}$ in 6 h.

Messenger (m)RNA turnover – mRNA degradation: mRNA degradation does not require any energy-requiring steps.[37]

Ribosome turnover – number of ribosome particles based on protein expression data: A typical human cell ribosomal protein constitutes 4 – 6% of the total protein mass and there are about 10^7 ribosomes/cell.[38] To estimate ribosome particle number based on protein expression data, the protein expression data of the small ribosomal subunit were averaged. Likewise, the protein expression data of the large ribosomal subunit were averaged. To obtain total ribosomal particle numbers the small and large subunits were averaged. We obtained 2.68 × 10^6 number of ribosomal particles in rPR, 2.11 ×

10^6 in cPR and 7.23×10^6 in RPE, which is in a similar range compared to a typical cell.

Ribosome turnover – number of ribosome particles based on weight of rRNA in cells: The number of NMPs in one ribosomal particle is 1800 (SSU) + 1800 + 5000 + 5000 (LSU) = 13600. Each NMP weights 321.47 g mol-1 (=Da). The weight of rRNA in one ribosomal particle is 13600×321.47 Da = 4.37×10^6 Da (1 Da = 1.66×10^{-24} g; this is the weight of single molecule where one mole of this molecule is on Dalton). To calculate the number of particles based on rRNA weight, the weight of rRNA based on molecular composition of cell mass was divided by 4.37×10^6 Da and further divided by 1.66×10^{-24}. Using this approach, a very similar number of ribosomal particles was calculated for rPR (9.11×10^5), cPR (9.11×10^5), and RPE (5.36×10^6). Interestingly, the approach bases on the expression of ribosomal protein subunits results in a slightly higher number of ribosomal particles; subunits have to be transported to the nucleus quickly as otherwise degraded in cytosol.

Ribosome turnover – transport of ribosomal protein subunits into nucleus: The ribosomal protein subunits need to be transported to the nucleus before being assembled into ribosomal particles that include the rRNA.[39] This requires RanGTP for nuclear import, and therefore hydrolysis of 1 GTP (1 ATP equivalent) per ribosomal protein subunit.[40]

The number of all ribosomal protein subunits in rPR is 2.33×10^8. With a half-life of 6 days (= 518400 s) the replacement rate is 2.25×10^2 ribosome protein subunits s^{-1}. With one ATP per subunit required, 2.25×10^2 ATP s^{-1} $cell^{-1}$ are needed in 24 h and in $4 \times 2.25 \times 10^2$ = of 9.00×10^2 ATP s^{-1} $cell^{-1}$ in 6 h.

The number of all ribosomal protein subunits in cPR is 1.85×10^8. With a half-life of 6 days (=518400 s) the replacement rate is 1.78×10^2 ribosome protein subunits s^{-1}. With one ATP per subunit required, 1.78×10^2 ATP s-1 cell-1 are needed in 24 h and in $4 \times 1.78 \times 10^2 = 7.12 \times 10^2$ ATP s^{-1} $cell^{-1}$ in 6 h.

The number of all ribosomal protein subunits in RPE is 6.41×10^8. With a half-life of 4.5 days (=388800 s) the replacement rate is 8.25×10^2 ribosome protein subunits s^{-1}. With 1 ATP per subunit required, 8.25×10^2 ATP s^{-1} $cell^{-1}$ are needed in 24 h and in $4 \times 8.25 \times 10^2 = 3.30 \times 10^3$ ATP s^{-1} $cell^{-1}$ in 6 h.

Ribosome turnover – production of rRNA in nucleus: 13600 NTPs are needed per ribosomal particle. For the energy budget, for each NTP 1 ATP is needed for helicase,[41] and 2 ATP as hydrolysis of NTP to NMP (= total of 3 ATP per NTP). The ribosomal particle numbers were taken based on calculations of the weight of rRNA.

The number of all ribosomal particles (based on rRNA) in rPR is 9.11×10^5, each containing 13600 NMPs (= NTPs that need to be replaced). With a half-life of 6 days (= 518400 s) the replacement rate is 1.19×10^4 NTPs s^{-1} $cell^{-1}$. 3 ATP are needed for every NTP, which results in 3.58×10^4 ATP s^{-1} $cell^{-1}$ in 24 h and in $4 \times 3.58 \times 10^4 = 1.43 \times 10^5$ ATP s^{-1} $cell^{-1}$ in 6 h.

The number of all ribosomal particles (based on rRNA) in cPR is 9.11×10^5, each containing 13600 NMPs (=NTPs that need to be replaced). With a half-life of 6 days (=518400 s) the replacement rate is 1.19×10^4 NTPs s^{-1} $cell^{-1}$. 3 ATP are needed for every NTP, which results in 3.58×10^4 ATP s^{-1} $cell^{-1}$ in 24 h and in $4 \times 3.58 \times 10^4 = 1.43 \times 10^5$ ATP s^{-1} $cell^{-1}$ in 6 h.

The number of all ribosomal particles (based on rRNA) in RPE is 5.36×10^6, each containing 13600 NMPs (=NTPs that need to be replaced). With a half-life of 4.5 days (=388800 s) the replacement rate is 9.37×10^4 NTPs s^{-1} $cell^{-1}$. 3 ATP are needed for every NTP, which results in 2.81×10^5 ATP s^{-1} $cell^{-1}$ in 24 h and in $4 \times 2.81 \times 10^5 = 1.12 \times 10^6$ ATP s^{-1} $cell^{-1}$ in 6 h.

Ribosome turnover – processing of small subunit: The small subunit, per particle, has one rRNA with 1800 NTPs. The processing of the small subunit has several energy-requiring steps.[39] We assumed the following requirements: GTPase needs 1 GTP (= 1 ATP) per rRNA molecule. Helicases need a total of 1 ATP for each NTP. For kinases, we assumed 3 ATP per rRNA molecule.

The number of all ribosomal particles (based on rRNA) in rPR is 9.11×10^5. The small subunit requires 1800 NTPs, which results, with a half-life of 6 days (=518400 s), in a demand of 1.58×10^3 NTPs s^{-1} $cell^{-1}$. There is one rRNA molecule in the SSU, which results, with a half-life of 6 days (=518400 s), in a replacement rate of 0.88 s^{-1}. With 1 ATP per NTP required and 4 ATP per rRNA molecule, the energy budget is $(1 \times 1.58 \times 10^3) + (4 \times 0.88) = 1.58 \times 10^3$ ATP s^{-1} $cell^{-1}$ in 24 h and in $4 \times 1.58 \times 10^3 = 6.32 \times 10^3$ ATP s^{-1} $cell^{-1}$ in 6 h.

The number of all ribosomal particles (based on rRNA) in cPR is 9.11×10^5. The small subunit requires 1800 NTPs, which results, with a half-life of 6 days (=518400 s), in a demand of 1.58×10^3 NTPs s^{-1} cell-1. There is one rRNA molecule in the SSU, which results, with a half-life of 6 days (= 518400 s), in a replacement rate of 0.88 s^{-1}. With 1 ATP per NTP required and 4 ATP per rRNA molecule, the energy budget is $(1 \times 1.58 \times 10^3) + (4 \times 0.88) = 1.58 \times 10^3$ ATP s^{-1} $cell^{-1}$ in 24 h and in $4 \times 1.58 \times 10^3 = 6.32 \times 10^3$ ATP s^{-1} $cell^{-1}$ in 6 h.

The number of all ribosomal particles (based on rRNA) in RPE is 5.36×10^6. The small subunit requires 1800 NTPs, which results, with a half-life of 4.5 days (=388800 s), in a demand of 1.24×10^4 NTPs s^{-1} $cell^{-1}$. There is one rRNA molecule in the SSU, which results, with a half-life of 4.5 days (=388800 s), in a replacement rate of 6.89 s-1. With 1 ATP per NTP required and 4 ATP per rRNA molecule, the energy budget is $(1 \times 1.24 \times 10^4) + (4 \times 6.89) = 1.24 \times 10^4$ ATP s^{-1} $cell^{-1}$ in 24 h and in $4 \times 1.24 \times 10^4 = 4.96 \times 10^4$ ATP s^{-1} $cell^{-1}$ in 6 h.

Ribosome turnover – processing of large subunit – nuclear steps: The large subunit, per particle, has three rRNA with a total of 11800 NTPs. The processing of the large subunit has several energy-requiring nuclear steps.[39] For helicases, assume a total of 1 ATP for each NTP. GTPases consume 2 GTP per subunit particle as acts as dimer. Rix7, an AAA type ATPase, for which 6 ATP per subunit particle were assumed as they act as hexamer. Rea1, an AAA type ATPase, for which also 6 ATP per subunit particle were assumed as act as hexamer.

The number of all ribosomal particles (based on rRNA) in rPR is 9.11×10^5. The large subunit requires 13600 NTPs, which results, with a half-life of 6 days (= 518400 s), in a demand of 1.04×10^4 NTPs s^{-1} $cell^{-1}$. There are three rRNA molecules in the LSU, which results, with a half-life of 6 days (= 518400 s), in a replacement rate of 2.64 s^{-1}. With 1 ATP per NTP required and 14 ATP per rRNA molecule, the energy budget is $(1 \text{ x } 1.04 \text{ x } 10^4) + (14 \text{ x } 2.64) = 1.04 \text{ x } 10^4$ ATP s^{-1} $cell^{-1}$ in 24 h and in $4 \times 1.04 \times 10^4 = 4.16 \times 10^4$ ATP s^{-1} $cell^{-1}$ in 6 h.

The number of all ribosomal particles (based on rRNA) in cPR is 9.11×10^5. The large subunit requires 13600 NTPs, which results, with a half-life of 6 days (= 518400 s), in a demand of 1.04×10^4 NTPs s^{-1} $cell^{-1}$. There are three rRNA molecules in the LSU, which results, with a half-life of 6 days (= 518400 s), in a replacement rate of 2.64 s^{-1}.

With 1 ATP per NTP required and 14 ATP per rRNA molecule, the energy budget is (1 × 1.04 × 10^4) + (14 × 2.64) = 1.04 × 10^4 ATP s^{-1} $cell^{-1}$ in 24 h and in 4 × 1.04 × 10^4 = 4.16 × 10^4 ATP s^{-1} $cell^{-1}$ in 6 h.

The number of all ribosomal particles (based on rRNA) in RPE is 5.36 × 10^6. The large subunit requires 13600 NTPs, which results, with a half-life of 4.5 days (=388800 s), in a demand of 8.14 × 10^4 NTPs s^{-1} $cell^{-1}$. There are three rRNA molecules in the LSU, which results, with a half-life of 4.5 days (=388800 s), in a replacement rate of 2.07 × 10^1 s^{-1} $cell^{-1}$. With 1 ATP per NTP required and 14 ATP per rRNA molecule, the energy budget is (1 × 8.14 × 10^4) + (14 × 2.07 × 10^1) = 8.16 × 10^4 ATP s^{-1} $cell^{-1}$ in 24 h and in 4 × 8.16 × 10^4 = 3.26 × 10^5 ATP s^{-1} $cell^{-1}$ in 6 h.

Ribosome turnover – processing of large subunit – cytoplasmic steps: The large subunit, per particle, has three rRNA with a total of 11800 NTPs. The processing of the large subunit has several energy-requiring cytoplasmic steps.[39] GTPase needs 1 GTP per subunit particle. Drg1, an AAA type ATPase, for which also 6 ATP per subunit particle were assumed as act as hexamer.

The number of all ribosomal particles (based on rRNA) in rPR is 9.11 x 10^5. There are three rRNA molecules in the LSU, which results, with a half-life of 6 days (= 518400 s), in a replacement rate of 2.64 s^{-1}. With 7 ATP per rRNA molecule required, the energy budget is (7 × 2.64) = 1.84 × 10^1 ATP s^{-1} $cell^{-1}$ in 24 h and in 4 × 1.84 × 10^1 = 7.36 × 10^1 ATP s^{-1} $cell^{-1}$ in 6 h.

The number of all ribosomal particles (based on rRNA) in cPR is 9.11 x 10^5. There are three rRNA molecules in the LSU, which results, with a half-life of 6 days (= 518400 s), in a replacement rate of 2.64 s^{-1}. With 7 ATP per rRNA molecule required, the energy budget is (7 × 2.64) = 1.84 × 10^1 ATP s^{-1} $cell^{-1}$ in 24 h and in 4 × 1.84 × 10^1 = 7.36 × 10^1 ATP s^{-1} $cell^{-1}$ in 6 h.

The number of all ribosomal particles (based on rRNA) in RPE is 5.36 x 10^6. There are three rRNA molecules in the LSU, which results, with a half-life of 4.5 days (=388800 s), in a replacement rate of 2.07 × 10^1 s^{-1} $cell^{-1}$. With 7 ATP per rRNA molecule required, the energy budget is (7 × 2.07 × 10^1) = 1.45 × 10^2 ATP s^{-1} $cell^{-1}$ in 24 h and in 4 × 1.45 × 10^2 = 5.08 × 10^2 ATP s^{-1} $cell^{-1}$ in 6 h.

Translation: Translation requires about 5 ATP per amino acid added to nascent polypeptide. The breakdown being: One ATP to AMP (= 2 ATP) for activation of tRNA,

one GTP -> GDP for EF-Tu transfer on the aminoacyl-tRNA to the A site, one GTP->GDP for the EF-G translocation of peptidyl-tRNA to the P site (Bionumbers ID: 101442). There is on average one more ATP to cover cost of error correction/proof reading giving a total cost of 5 ATP per peptide bond (Bionumbers ID: 107782).

Based on a turnover of 10 days (= 240 h) in outer segments and a protein abundance of 3.05×10^8 in rPR cells, this results in a demand of 3.53×10^2 proteins s^{-1} $cell^{-1}$ = 1.32×10^5 aa s^{-1} $cell^{-1}$. Based on a half-life of 5.4 days (= 129.6 h) in the rest of the rPR and a protein abundance of 1.04×10^9, this results in a demand (= replacement rate) of 1.11×10^3 proteins s^{-1} $cell^{-1}$ = 4.18×10^5 aa s^{-1} $cell^{-1}$. This results in a total protein replacement rate of 1.47×10^3 proteins s^{-1} $cell^{-1}$ and a total amino acid requirement for protein replacement of 5.50×10^5 aa s^{-1} $cell^{-1}$. [Note: based on ribosome abundance of 2.86×10^6 particles and translation speed of 20 aa s^{-1} (=5.27×10^7 s^{-1} $cell^{-1}$), the ribosome can deliver ~ 137-times more of what is needed based on demand]. Based on a total protein replacement rate of 1.47×10^3 proteins s^{-1} rod $cell^{-1}$ and a total amino acid requirement of 5.50×10^5 aa s^{-1} $cell^{-1}$, this results in an energy budget of $(5 \times 5.50 \times 10^5) + (4 \times 1.47 \times 10^3) = 2.76 \times 10^6$ ATP s^{-1} $cell^{-1}$ in 24 h and in $4 \times 2.76 \times 10^6 = 1.10 \times 10^7$ ATP s^{-1} $cell^{-1}$ in 6 h.

Based on a half-life of 5.4 days (= 129.6 h) and a protein abundance of 1.35×10^9 in cPR cells this results in a demand (= replacement rate) of 1.45×10^3 proteins s^{-1} $cell^{-1}$ = 5.43×10^5 aa s^{-1} $cell^{-1}$. [Note: based on ribosome abundance of 2.26×10^6 particles and translation speed of 20 aa s^{-1} (= 4.52×10^7 s^{-1} $cell^{-1}$), the ribosome can deliver ~ 83-times more of what is needed based on demand.] Based on demand, this results in an energy budget for translation of $(5 \times 5.43 \times 10^5) + (4 \times 1.45 \times 10^3) = 2.72 \times 10^6$ ATP s^{-1} $cell^{-1}$ in 24 h and in $4 \times 2.72 \times 10^6 = 1.09 \times 10^7$ ATP s^{-1} $cell^{-1}$ in 6 h.

Based on a half-life of 3 days (= 72 h) and a protein abundance of 8.96×10^9 in RPE cells, this results in a demand (= replacement rate) of 1.73×10^4 proteins s^{-1} $cell^{-1}$ = 6.48×10^6 aa s^{-1} $cell^{-1}$. [Note: based on ribosome abundance of 7.87×10^6 particles and translation speed of 20 aa s^{-1} (=1.57×10^8 s^{-1} $cell^{-1}$), the ribosome can deliver ~ 24-times more of what is needed based on demand). Based on demand (aka replacement rates of 1.73×10^4 proteins s^{-1} $cell^{-1}$ = $6.48 \text{ x } 10^6$ aa s^{-1} $cell^{-1}$), this results in $(5 \times 6.48 \times 10^6) + (4 \times 1.73 \times 10^4) = 3.25 \times 10^7$ ATP s^{-1} $cell^{-1}$ in 24 h and in $4 \times 3.25 \text{ x } 10^7 = 1.30 \times 10^8$ ATP s^{-1} $cell^{-1}$ in 6 h.

Protein chaperone folding: 5 ATP are needed per protein for chaperone-mediated folding.[42]

Based on a replacement rate of 1.47×10^3 proteins s^{-1} $cell^{-1}$ in rPR, this results in an energy budget of $5 \times 1.47 \times 10^3 = 7.34 \times 10^3$ ATP s^{-1} $cell^{-1}$ in 24 h and in $4 \times 7.34 \times 10^3 = 2.94 \times 10^4$ ATP s^{-1} $cell^{-1}$ in 6 h.

Based on a replacement rate of 1.45×10^3 proteins s^{-1} $cell^{-1}$ in cPR, this results in an energy budget of $5 \times 1.45 \times 10^3 = 7.23 \times 10^3$ ATP s^{-1} $cell^{-1}$ in 24 h and in $4 \times 7.23 \times 10^3 = 2.89 \times 10^4$ ATP s^{-1} $cell^{-1}$ in 6 h.

Based on a replacement rate of 1.73×10^4 proteins s^{-1} $cell^{-1}$ in RPE, this results in an energy budget of $5 \times 1.73 \times 10^4 = 8.64 \times 10^4$ ATP s^{-1} $cell^{-1}$ in 24 h and in $4 \times 8.64 \times 10^4 = 3.46 \times 10^5$ ATP s^{-1} $cell^{-1}$ in 6 h.

Protein posttranslational modifications (PTMs): We assume that posttranslational modifications are dynamic and happen throughout the 24-h light and dark cycle.

Protein posttranslational modifications (PTMs) – Phosphorylation: There are a total 230000 phosphorylation sites.[43] Based on 19300 proteins in the human proteome, this results in an average of 230000/19300 = 12 phosphorylation sites per protein, where 1 ATP per site (by upstream kinase) is needed.

Based on a replacement rate of 1.47×10^3 proteins s^{-1} $cell^{-1}$ in rPR, this results in an energy budget of $12 \times 1 \times 1.47 \times 10^3 = 1.76 \times 10^4$ ATP s^{-1} $cell^{-1}$.

Based on a replacement rate of 1.45×10^3 proteins s^{-1} $cell^{-1}$ in cPR, this results in an energy budget of $12 \times 1 \times 1.45 \times 10^3 = 1.74 \times 10^4$ ATP s^{-1} $cell^{-1}$.

Based on a replacement rate of 1.73×10^4 proteins s^{-1} $cell^{-1}$ in RPE, this results in an energy budget of $12 \times 1 \times 1.73 \times 10^4 = 2.07 \times 10^5$ ATP s^{-1} $cell^{-1}$.

Protein posttranslational modifications (PTMs) – Glycosylation: 60% of proteins are glycosylated.[44] Synthesis of glycans requires (https://en.wikipedia.org/wiki/N-linked_glycosylation) attachment of an oligosaccharide, a carbohydrate consisting of several sugar molecules, where 2 ATP are required. The average number of glycosylation per protein is 2294/ 1132 = 2.[45]

Based on a replacement rate of 1.47×10^3 proteins s^{-1} $cell^{-1}$ in rPR, this results in an energy budget of $2 \times 2 \times 1.47 \times 10^3 = 5.87 \times 10^3$ ATP s^{-1} $cell^{-1}$.

Based on a replacement rate of 1.45×10^3 proteins s^{-1} $cell^{-1}$ in cPR, this results in an energy budget of $2 \times 2 \times 1.45 \times 10^3 = 5.79 \times 10^3$ ATP s^{-1} $cell^{-1}$.

Based on a replacement rate of 1.73×10^4 proteins s^{-1} $cell^{-1}$ in RPE, this results in an energy budget of $2 \times 2 \times 1.73 \times 10^4 = 6.91 \times 10^4$ ATP s^{-1} $cell^{-1}$.

Protein posttranslational modifications (PTMs) – Acetylation: Acetylation requires addition of Acetyl-CoA. To consider Acetyl-CoA in the energy budget, we consider that Acetyl-CoA can produce 11 ATP and 1 GTP per acetyl group (https://en.wikipedia.org/wiki/Acetyl-CoA). There are 22863 number of acetylation sites in 19300 proteins (https://awi.cuhk.edu.cn/dbPTM/download.php). This corresponds to 1.18 acetylation sites per protein.

Based on a replacement rate of 1.47×10^3 proteins s^{-1} $cell^{-1}$ in rPR, this results in an energy budget of $1.18 \times 12 \times 1.47 \times 10^3 = 2.08 \times 10^4$ ATP s^{-1} $cell^{-1}$.

Based on a replacement rate of 1.45×10^3 proteins s^{-1} $cell^{-1}$ in cPR, this results in an energy budget of $1.18 \times 12 \times 1.45 \times 10^3 = 2.05 \times 10^4$ ATP s^{-1} $cell^{-1}$.

Based on a replacement rate of 1.73×10^4 proteins s^{-1} $cell^{-1}$ in RPE, this results in an energy budget of $1.18 \times 12 \times 1.73 \times 10^4 = 2.45 \times 10^5$ ATP s^{-1} $cell^{-1}$.

Protein posttranslational modifications (PTMs) – Ubiquitination: Ubiquitination requires 1 ATP for E1 activation. There are 30407 number of ubiquitination sites in 19300 proteins (https://awi.cuhk.edu.cn/dbPTM/download.php). This corresponds to 1.58 ubiquitination sites per protein.

Based on a replacement rate of 1.47×10^3 proteins s^{-1} $cell^{-1}$ in rPR, this results in an energy budget of $1 \times 1.58 \times 1.47 \times 10^3 = 2.32 \times 10^3$ ATP s^{-1} $cell^{-1}$.

Based on a replacement rate of 1.45×10^3 proteins s^{-1} $cell^{-1}$ in cPR, this results in an energy budget of $1 \times 1.58 \times 1.45 \times 10^3 = 2.29 \times 10^3$ ATP s^{-1} $cell^{-1}$.

Based on a replacement rate of 1.73×10^4 proteins s^{-1} $cell^{-1}$ in RPE, this results in an energy budget of $1 \times 1.58 \times 1.73 \times 10^4 = 2.73 \times 10^4$ ATP s^{-1} $cell^{-1}$.

Protein degradation via proteasome: We initially assumed that 50% of the proteins are degraded via the proteasome and 50% via the lysosome. On average, 100 ATP are

needed to degrade one protein.[46] To see if the assumption of 50% degradation via the proteasome is a reasonable assumption, we later compared it to the abundance of the proteasome and its catalytic activity of ~1 s^{-1}.[47]

There is no proteasomal degradation in the outer segments of rPR cells as proteins are phagocytosed by RPE cells. Protein turnover in the remaining part of the rPR is $1.11 \times 10^3/2$ proteins s^{-1} $cell^{-1}$, which results in a budget of 5.57×10^4 ATP s^{-1} $cell^{-1}$. To see how this relates to the proteasome activity, we used the enzyme abundance (1.43×10^5 molecules per rPR cell) together with k_{cat} (1 ATP s^{-1}), which results in 1.43×10^5 ATP s^{-1} $cell^{-1}$, which is similar (2.5-fold higher) to what is needed by demand. We used the average of 9.94×10^4 ATP s^{-1} $cell^{-1}$. As the capacity of the proteasome is already at the maximum, we assume that proteasomal activity we assumed that this process is happening throughout the 24 night and dark cycles.

Based on a replacement rate of 1.45×10^3 /2 proteins (50% of the proteins) s^{-1} $cell^{-1}$ in cPR, this results in an energy budget of 7.23×10^4 ATP s^{-1} $cell^{-1}$. To see how this relates to the proteasome activity, we used the enzyme abundance (9.59×10^4 molecules per cPR cell) together with k_{cat} (1 ATP s^{-1}), which results in 9.59×10^4 ATP s^{-1} $cell^{-1}$, which is similar (1.3-fold higher) to what is needed by demand. We used the average of 8.41×10^4 ATP s^{-1} $cell^{-1}$.

Based on a replacement rate of 1.73×10^4 /2 proteins s^{-1} $cell^{-1}$ in RPE, this results in an energy budget of 8.64×10^5 ATP s^{-1} $cell^{-1}$. To see how this relates to the proteasome activity, we used the enzyme abundance ($5.87 \text{ x } 10^5$ molecules per RPE cell) together with k_{cat} (1 ATP s^{-1}), which results in 5.87×10^5 ATP s^{-1} $cell^{-1}$, which is 1.5-fold lower compared to what is needed by demand. We used the average of 7.26×10^5 ATP s^{-1} $cell^{-1}$.

Protein degradation via lysosome: Each peptide bond requires 1 H^+. The stoichiometry for how many protons V-ATPase pumps per ATP is very variable[48] with figures varying from 2, 3.3 and 3.3 to 5.[49-51] V-ATPases are thought to act in a similar manner to F-ATPases where the accepted stoichiometry is 3 to 4 protons/ATP.[52] A couple of reviews quote the figure of 2 but without reference. Here, we assumed a value of 2.65 for the stoichiometry of V-ATPase. To see how this relates to the lysosomal activity, we used the enzyme abundances together with k_{cat} (10 s^{-1} rotations).[53]

There is no lysosomal degradation in the outer segments of rPR cells as proteins are phagocytosed by RPE cells. The replacement rate in the remaining part of the rPR is 4.18×10^5 aa s^{-1} $cell^{-1}$, which results in a budget of $4.18 \times 10^5/2/ 2.65 = 1.04 \times 10^5$ ATP s^{-1} $cell^{-1}$. To see how this relates to the lysosomal activity, we used the enzyme abundance (2.44×10^5 molecules per rPR cell) together with k_{cat} (10 s^{-1} rotations), which results in 2.44×10^6 ATP s^{-1} $cell^{-1}$, which is 24-fold higher to what is needed by demand. We assumed that lysosomal activity is highest during the 1st 6 h of day. As this is a process estimated from the demand, the required energy is per 24 h and if applied to 6 h, this value has to be multiplied by 4, resulting in a budget of $4 \times 1.04 \times 10^5 = 4.16 \times 10^5$ ATP s^{-1} $cell^{-1}$ in 6 h.

Based on a replacement rate of 5.43×10^5 aa s^{-1} $cell^{-1}$ in cPR, this results in an energy budget of $5.43 \times 10^5 / 2.65 /2 = 1.02 \times 10^5$ ATP s^{-1} $cell^{-1}$. To see how this relates to the lysosomal activity, we used the enzyme abundance (2.28×10^5 molecules per cPR cell) together with k_{cat}, which results in 2.28×10^6 ATP s^{-1} $cell^{-1}$, which is 22-fold higher to what is needed by demand. The energy required in 6 h is $4 \times 1.02 \times 10^5 = 4.08 \times 10^5$ ATP s^{-1} $cell^{-1}$.

Based on a replacement rate of 6.48×10^6 aa s^{-1} $cell^{-1}$ in RPE, this results in an energy budget of $6.48 \times 10^6 / 2.65/ 2 = 1.22 \times 10^6$ ATP s^{-1} $cell^{-1}$. To see how this relates to the lysosomal activity, we used the enzyme abundance (1.25×10^6 molecules per RPE cell) together with k_{cat}, which results in 1.25×10^7 ATP s^{-1} $cell^{-1}$, which is 10-fold higher to what is needed by demand. The energy required in 6 h is $4 \times 1.22 \times 10^6 = 4.88 \times 10^6$ ATP s^{-1} $cell^{-1}$.

LIPID TURNOVER

Lipid membrane production: The outer segment contains equal amounts of lipid and protein by weight.[54] The mammalian rod has a length of 20 to 30 µm and a diameter of 1.2 to 2.0 µm. Each photoreceptor disc is about 10 nm thick and stacked at a spacing of about 25 nm so that gives about 1000 discs/outer segment. This gives a surface area of the outer segment and discs of around 6.3×10^3 μm^2. About half is protein and of the remainder, 30% is cholesterol.

Cholesterol production: Cholesterol production was only considered in outer segments of the rod photoreceptor. In the remaining part of the rPR, in cPR, and in RPE we

assumed that cholesterol will be largely recycled and be only a neglectable energy term.

There are about 5×10^6 lipid molecules per square micrometer of plasma membrane (and having allowed for 50% being protein) giving a $5 \times 10^6 \times 6.3 \times 10^3 = 3.15 \times 10^{10}$ lipid molecules/rod outer segment. [Note, this number is in agreement (= below) with the total number of lipid molecules of 2.79×10^{10} in rPR calculated based on the molecular composition of a standard cell based on weight, Supplementary Table S1].

There are two terms here:

- Recycling term
- Production

The retina has the capacity to synthesize cholesterol with rapid incorporation of labelled acetate[55,56] or labelled mevalonolactone[57] into cholesterol which, among other fates, is exported along the optic nerve. In the frog retina, labelled squalene was the major product following labelled acetate and is destined for rod outer segment synthesis[57] but calculations suggest an insufficient amount to account for the whole cholesterol requirement.[58] An estimate for cholesterol requirements for the rod is in the region of 35 to 50 pmol/retina/hour compared to an observed rate of 3.4 pmol/retina/hour.[58] So, there is recycling. Each day, 10% of lipids are lost. With respect to cholesterol, 5/6 are recycled and 1/6 is shed.[58,59] To synthesize one cholesterol requires 18 Acetyl-CoA, 18 ATP and 5 NADPH molecules, which corresponds to $(18 \times 12) + 18 + (5 \times 2.5) = 246.5$ ATP.

There are 3.15×10^{10} lipid molecules in rod outer segments, of which 1/10 are turned over per day (= 3.15×10^9 lipid molecules day^{-1} = 3.65×10^4 lipid molecules s^{-1}) of which 1/6 is cholesterol. To synthesize one cholesterol requires 18 Acetyl-CoA, 18 ATP and 5 NADPH molecules, which corresponds to (18×12 for Acetyl-CoA) + 18 + (5×2.5 for average yield of ATP from NADPH) = 246.5 ATP. This results in a budget of 4.25×10^5 ATP s^{-1} cell^{-1}. This process is assumed to happen over the 2nd 6 h period of day. As this is a process estimated from the demand, the required energy is per 24 h and if applied to 6 h, this value has to be multiplied by 4, resulting in a budget of $4 \times 4.25 \times 10^5 = 1.70 \times 10^6$ ATP s^{-1} cell^{-1} in 6 h.

<u>Phospholipid turnover</u>: A complete cycle of breakdown of triacylglycerols (TG) into glycerol and free fatty acids (FFA) and regeneration of TG required 7 ATP (2 per acyl

group and 1 for phosphorylation of glycerol).[60] As most membrane lipids have one acyl chain replaced by a head group (e.g. phosphate, choline), we considered 5 ATP per phospholipid turnover cycle. Only synthesis requires energy and was assumed that synthesis is taking place in the 2nd 6 h of day.

Knowing the total number of phospholipids per cell and assuming the half-life of lipids in the ER of 4 days[61] driving the turnover cycle, the energy required can be calculated.

In rPR the sum of phospholipids is 2.08 x 10^{10} molecules (Supplementary Table S1). With a half-life of 4 days, this results in a replacement rate of 3.01 x 10^4 phospholipids s^{-1} $cell^{-1}$. With 5 ATP per cycle required, the budget is 1.5 x 10^5 ATP s^{-1} $cell^{-1}$ in 24 h, which is 4 * 1.5 x 10^5 = 6 x 10^5 ATP s^{-1} $cell^{-1}$ (as estimated from demand).

In cPR the sum of phospholipids is 2.08×10^{10} molecules (Supplementary Table S1). With a half-life of 4 days, this results in a replacement rate of 3.01×10^4 phospholipids s^{-1} $cell^{-1}$. With 5 ATP per cycle required, the budget is 1.5×10^5 ATP s^{-1} $cell^{-1}$ in 24 h, which is $4 \times 1.5 \times 10^5 = 6 \times 10^5$ ATP s^{-1} $cell^{-1}$ (as estimated from demand).

In RPE the sum of phospholipids is 1.31×10^{11} molecules (Supplementary Table S1). With a half-life of 4 days, this results in a replacement rate of 1.9×10^5 phospholipids s^{-1} $cell^{-1}$. With 5 ATP per cycle required, the budget is 9.48×10^5 ATP s^{-1} $cell^{-1}$ in 24 h, which is $4 \times 9.48 \times 10^5 = 3.79 \times 10^6$ ATP s^{-1} $cell^{-1}$ (as estimated from demand).

<u>Maintaining cell membrane asymmetry (flippases)</u>: To calculate the energy required for maintaining cell membrane asymmetry, we summed the abundance of all flippases (ATP8A2, ATP8A1, ATP8B1, ATP8B1, ATP8B3, ATP11A, ATP11C) and multiplied by k_{cat} (= 0.5 s^{-1}).[62] We assume that flippase are continuously active over 24 h.

The sum of flippases in rPR is 1.28×10^5, which results in an energy budget of $1.28 \times 10^5 \times 0.5 = 6.40 \times 10^5$ ATP s^{-1} $cell^{-1}$.

The sum of flippases in cPR is 2.44 x 10^5, which results in an energy budget of $2.44 \times 10^5 \times 0.5 = 1.22 \times 10^5$ ATP s^{-1} $cell^{-1}$.

The sum of flippases in RPE is 7.93×10^5, which results in an energy budget of $7.93 \times 10^5 \times 0.5 = 3.97 \times 10^5$ ATP s^{-1} $cell^{-1}$.

INTRACELLULAR ION TRANSPORT

Lysosomes: The k_{cat} of the vacuolar H+ ATPase is 10 s^{-1} rotations.[53] As we have calculated the demand of lysosomal V-ATPase for protein degradation, the remaining capacity is available for other lysosomal functions and can be calculated from maximum capacity minus requirement of protein degradation by demand.

With a maximal lysosomal V-ATPase capacity of 2.44 × 10^6 ATP s^{-1} $cell^{-1}$ in rPR cells and a demand for protein degradation of 1.04 × 10^5 ATP s^{-1} $cell^{-1}$, this results in a remaining capacity of 2.34 × 10^6 ATP s^{-1} $cell^{-1}$.

With a maximal lysosomal V-ATPase capacity of 2.28 × 10^6 ATP s^{-1} $cell^{-1}$ in cPR cells and a demand for protein degradation of 1.02 × 10^5 ATP s^{-1} $cell^{-1}$, this results in a remaining capacity of 2.18 × 10^6 ATP s^{-1} $cell^{-1}$.

With a maximal lysosomal V-ATPase capacity of 1.25 × 10^7 ATP s^{-1} $cell^{-1}$ in RPE cells and a demand for protein degradation of 1.22 × 10^6 ATP s^{-1} $cell^{-1}$, this results in a remaining capacity of 1.13 × 10^7 ATP s^{-1} $cell^{-1}$.

Endoplasmic reticulum and golgi: The catalytic activity of the sarcoplasmic/endoplasmic reticulum calcium ATPase (ATP hydrolysis) is 36.6 micromol Pi per mg protein in 60 min.[63] The energy budget can be worked out by first calculating the amount of enzyme (sum of ATP2A1, ATP2A2, and ATP2A3) in each cell type in mg. This results in a molar ATP amount per time, which can be converted into molecules of ATP s^{-1} $cell^{-1}$. We assume that this is a basic homeostatic processes and happening continuously over the 24 h.

The sum of ATP2A1, ATP2A2, and ATP2A3 in rPR cells is 2.1 × 10^{-7} mol L^{-1}, which corresponds to 1.18 x 10^{-11} mg enzyme per cell. This corresponds to a budget of 7.24 × 10^4 ATP s^{-1} $cell^{-1}$.

The sum of ATP2A1, ATP2A2, and ATP2A3 in cPR cells is 3.42 × 10^{-7} mol L^{-1}, which corresponds to 1.93 × 10^{-11} mg enzyme per cell. This corresponds to a budget of 1.18 × 10^5 ATP s^{-1} $cell^{-1}$.

The sum of ATP2A1, ATP2A2, and ATP2A3 in RPE cells is 1.09 × 10^{-6} mol L^{-1}, which corresponds to 3.89 × 10^{-10} mg enzyme per cell. This corresponds to a budget of 2.38 × 10^6 ATP s^{-1} $cell^{-1}$.

DNA REPAIR

Types of DNA damage and scale of problem: There are four types of DNA damage classes: (i) Base modification; (ii) base loss; (iii) base crosslink, and (iv) strand breaks.

Examples for base modifications are base mismatches (caused by replication errors and does not apply to non-dividing cells in the retina), oxidative damage (caused by endogenous reactive oxygen and nitrogen species, ROS, RNS), alkylation/deamination (caused by metabolic by-products), and bulky adduct formations (caused by exogenous mutagens/carcinogens). Oxidative damage occurs at rate of 1×10^4 events $cell^{-1}$ day^{-1}.[64] Alkylation and deamination occurs at a rate of 1.1×10^3 events $cell^{-1}$ day^{-1}.[65] Bulky adduct formation occurs at a rate of 1.1×10^3 events $cell^{-1}$ day^{-1}.[65]

Examples for base losses are depurinations and depyrimidations (caused by intrinsic hydrolysis). Indeed, glycosyl bonds are intrinsically labile – particularly for purines. It is estimated that 2000 to 10,000 purine bases turn over every day in a human cell.[66] The rate is × 20 less for pyrimidines. Another estimate was 5,000 – 20,000 abasic DNA molecules at steady state with the brain have the highest proportion.[67] A further source suggests a rate of 1×10^4 events $cell^{-1}$ day^{-1} for depurination and 1×10^2 events $cell^{-1}$ day^{-1} for depyrimidation.[65]

Examples for base crosslinks are photodimers (caused by UV light) and intra-and interstrand crosslinks (caused by exogenous agents such as chemotherapeutics[65]; not considered here). They occur at a rate of 1×10^2 events $cell^{-1}$ day^{-1}.[65] We assumed that the rate is similar as in skin, which is justifiable as although stronger light intensity because of lens properties, this is compensated by filtering of light by the cornea.

Examples for strand breaks are single strand breaks (SSB) and double strand breaks (DSB) (caused by ionizing radiation). The rate of production of single strand breaks is 1000/cell/hour (= 2.4×10^4 events $cell^{-1}$ day^{-1}) and double strand breaks are 100 fold less (2.4×10^2 events $cell^{-1}$ day^{-1}).[68] Another source suggests that SSB are at a rate of 1×10^4 events $cell^{-1}$ day^{-1} and DSB at 1.03×10^1 events $cell^{-1}$ day^{-1}.[65]

Averaging across the different sources results in a total DNA damage of 3.75×10^4 events $cell^{-1}$ day^{-1}. This is close to a suggested number of 1×10^4 DNA lesion per day in each of our body cells.[69]

Repair mechanisms and energy required: There are five types of DNA repair mechanisms: (i) Mismatch Repair (MMR), (ii) Base Excision Repair (BER), (iii)

Nucleotide Excision Repair (NER), (iv) Non-Homologous End-Joining (NHEJ), and (v) Homologous Recombination (HR).

Oxidative damage (1 × 10^4 events day^{-1} $cell^{-1}$) is repaired through BER (non-bulky lesions) and NER (bulky lesions).[70] We assumed that half of the damage is repaired via BER and the other half via NER. We assumed for BER that on average 5 nucleotides need to be replaced of which one is damaged that needs to be synthetized de-novo. However, for helicases and other enzymes to act, we assumed that a stretch of 25 nucleotides needs to unwind (1 ATP per nucleotide for helicase). De novo synthesis of dNTPs requires 1 Glucose and (depending on type of dNTP either 10, 10, 7 or 8 =) 8.75 ATP equivalents.[71] 2 ATP per dNTP are needed for polymerization and 1 per dNTP for DNA ligase. This results in a budget of (25 × 1 ATP for helicase) + (5 × 2 ATP for polymerisation) + (5 × 1 ATP for ligase) + (8.75 ATP for de-novo dNTP) + (1 Glucose for de-novo dNTP) per lesion = 48.75 ATP + 1 Glucose per lesion, and a total of 2.82 ATP s^{-1} $cell^{-1}$ and 5.79 × 10^{-2} Glucose s^{-1} $cell^{-1}$ for the BER part of oxidative damage correction.

For oxidative damage correction via NER, ~30 nucleotides need to be replaced,[72] of which we assumed 5 dNTPs need to be synthetized de-novo. As NER repairs bulkier lesions, we assumed that a stretch of 50 nucleotides needs to unwind. This results in a budget of (50 × 1 ATP for helicase) + (30 × 2 ATP for polymerisation) + (30 × 1 ATP for ligase) + (5 × 8.75 ATP for de-novo dNTP) + (5 Glucose for de-novo dNTP) per lesion = 183.75 ATP + 5 Glucose per lesion, and a total of 10.6 ATP s^{-1} $cell^{-1}$ and 2.89 × 10^{-1} Glucose s^{-1} $cell^{-1}$ for the NER part of oxidative damage correction.

The correction of alkylation/ deamination, depurination, depyrimidation and SSB damage (total of 2.63 × 10^4 events day^{-1} $cell^{-1}$) involves BER. Similarly, as described before, the energy budget for BER is 48.75 ATP + 1 Glucose per lesion. This results in a budget of 14.8 ATP s^{-1} $cell^{-1}$ and 3.04 × 10^{-1} Glucose s^{-1} $cell^{-1}$.

Bulky adduct formations (1.1 × 10^3 events day^{-1} $cell^{-1}$) require NER for correction, which requires (see above) 183.75 ATP + 5 Glucose per lesion. This results in a budget of 2.34 ATP s^{-1} $cell^{-1}$ and 1.27 × 10^{-2} Glucose s^{-1} $cell^{-1}$.

DSB (1.25 × 10^2 events day^{-1} $cell^{-1}$) are corrected via NHEJ and HR. It was previous estimated that the repair of a one double strand break requires 1 × 10^4 ATP (Bionumbers ID: 112972). This results in a budget of 14.5 ATP s^{-1} $cell^{-1}$.

This results in a total energy requirement for DNA repair of 45.06 ATP s^{-1} $cell^{-1}$ and 0.66 Glucose s^{-1} $cell^{-1}$. We considered it to be the same in all cell types.

GLUTAMATE RECYCLING OF SYNAPTIC VESICLES

In rod PR: This is happening in the dark. Each synaptic vesicle contains an estimated 3640 glutamate molecules with a concentration inside the vesicle of 210 mM.[73] The recycling of glutamate requires a number of steps, the energetic of which are known and this allows one to make a calculation for how much energy is required per glutamate released:

Step 1 – Glutamate uptake: Glutamate transport from the synaptic cleft (into the Mueller cell) by EAAT1, 2, and 3 (SLC1A3, SLC1A2, SLC1A1) is coupled to the co-transport of three sodium and one proton (and the counter transport of one K^+) which have to be pumped out [H^+(out) + K^+(in) + L-glutamate(out) + 3 Na^+(out) -> H^+(in) + K^+(out) + L-glutamate(in) + 3 Na^+(in)].[74] Removal of 3 Na^+ from inside (the Mueller cell) back to outside requires sodium-potassium ATPase (ATP1A1, ATP1A2, ATP1A3, ATP1A4) [ATP + H_2O + K^+(out) + Na^+(in) = ADP + H^+ + K^+(in) + Na^+(out) + phosphate]. Hence, this is 1 ATP per glutamate molecule for 3 Na^+. In addition, one proton is taken up and this is removed by exchange with sodium (using sodium-hydrogen antiporter; SLC9A3, SLC9A1, SLC9A2, SLC9A4, SLC9A5, SLC9A6, SLC9A7, SLC9A8, SLC9A9) which has to be pumped (using sodium-potassium ATPase). This is 1/3 of an ATP. In total, step 1 requires 1.33 ATP per glutamate molecule (the energy required for this comes from the Mueller cell).

Step 2 – Conversion of Glutamate to Glutamine: This step happens in Mueller cells using Glutamine synthetase (GLUL) [ATP + L-glutamate + $NH4^+$ = ADP + H^+ + L-glutamine + phosphate]. One ATP is required per glutamate molecule in Mueller cells.

Step 3 – Release of Glutamine, uptake in rPR (or cPR, see later) and conversion to Glutamate: The steps are energetically neutral.

Step 4 – uptake of glutamate into vesicles: Glutamate is loaded into synaptic vesicles via the vesicular glutamate transporter (VGLUT1/SLC17A7, VGLUT2/SLC17A6, VGLUT3/SLC17A8) that utilizes a proton gradient, maintained by an ATP-dependent proton pump (V-ATPase). This is powered by the vesicular H^+-ATPase and, assuming the same H^+/ATP stoichiometry as for mitochondrial F-type ATPase, uses one-third of

an ATP molecule. However, there is a leak from these vesicles that needs to be counteracted increasing this figure to 1.14 ATP/glutamate.[75]

Total energy associated to synaptic vesicles: Considering steps 1 to 4 described above, this gives a total of 3.47 ATP/glutamate (2.33 in the Mueller cell and 1.14 in rPR/ cPR) and at 4000 glutamate per vesicle, an estimate of 11,000 ATP per vesicle released (calculation based on Attwell[76]). The release rate for rods is estimated at 127 vesicles per terminal under dark conditions. There is considerable species variation and this figure is for the salamander and for the Gecko, it is 35 vesicles per terminal.[77] This gives a figure per rod of 5.79×10^5 ATP/rod/second and an accompanying 1.18×10^6 ATP/second occurring in the Muller cells.

In cone PR: This is similar to the calculation for rods and there is quantitative analysis available for the cone ribbon synapse.[78]

Energy associated to synaptic vesicles: Considering steps 1-4 described above (section 2.2.), there are 2.33 ATP/glutamate required in the Mueller cell and 1.14 ATP/glutamate the cPR. There are 4000 glutamate molecules per vesicle. In the dark, the release is ~500 vesicles per second[77] consistent with 13 ribbons per synapse and a release rate of ~40 vesicles per ribbon. (It should be noted that there is considerable species variation.) This gives a figure per cone of 2.28×10^6 ATP/cone/second and an accompanying 4.66×10^6 ATP/second occurring in the Muller cells.

VISUAL TRANSDUCTION RODS

Step 1 – Light-induced Rhodopsin activation: Rhodopsin is present at high concentration in rod outer segments at an estimated 3 mM. Rhodopsin is densely packed in the disc membranes and forms rows of dimers.[79] A typical activation scale ranges from 0 active rhodopsin molecules (=Rh*)/rod/s to 10000 Rh*/rod/s.[5] To obtain an energy budget for visual transduction at indoor light levels, we assumed an activation of 600 Rh*/rod/s based on calibration to human photonic luminance (Candela/m^2),[80] by assuming indoors to correspond to 10^2 Candela/m^2, 10^{-6} Candela/m^2 to correspond to 0 Rh*/rod/s and 10^6 Candela/m^2 to correspond to 10000 Rh*/rod/s.

Step 2 – Transducin activation: One Rh* can activate several transducin complexes thereby allowing an amplification step. Out of several parameters, 20 per Rh*, 60-80, and 1250,[5,81,82] we selected 70 transducin activations per active rhodopsin. Transducin is a G-protein and hydrolysis GTP to GTP, which must be recycled at a cost of 1 ATP, which results in a energy budget of 70 × 600 = 4.20 × 10^4 ATP $cell^{-1}$ s^{-1}.

Step 3 – PDEdelta activation and cGMP hydrolysis: Phosphodiesterase (PDE) is the target of activated transducin. It takes two transducin subunits to bind to PDE to become active. The first site is of high affinity but has little effect on PDE activity. It requires the binding of a second and low affinity to activate PDE.[83] This double activation means that the number of activated PDE is about a quarter to a third of the number of transducin molecules. So a single activated rhodopsin (a single photon response) will generate about 25 activated PDE.[84] Each active PDE hydrolyses 100 cGMP molecules to GMP (assuming a 50 ms activation with PDE activity of 4000/s), where 2 ATP are needed per hydrolysis step (to recycle back to GTP to be available as guanylate cyclase substrate under dark conditions). This results in an energy budget for this step of 25 × 100 × 2 = 1.00 × 10^6 ATP $cell^{-1}$ s^{-1}.

Step 4 – Phosphorylation of Rhodopsin by Rhodopsin kinase: We assumed on average three phosphorylations by rhodopsin kinase per active rhodopsin,[85] each requiring 1 ATP. This results in an energy budget of 3 × 600 = 1.80 × 10^3 ATP $cell^{-1}$ s^{-1}.

Step 5 – Retinal recycling in rPR: The reduction of retinal to retinol requires NADPH. The likely source of this is the pentose phosphate pathway where one glucose is consumed for the production of 12 NADPH. The rate of NADPH production in bovine rod outer segments under optimal conditions is 40 nmol/mim/mg ROS protein.[86] The role of ABCA4 protein is assumed to be involved in the transport of retinol. In particular, it may act as an outwardly directed flippase for N-retinylidene-phosphatidylethanolamine and accordingly have a role in the removal of all trans-retinal. To calculate the energy required for the visual cycle in rPR, we assumed 0.3 ATP per Rh* for ABCA4 in rod cells. In addition, reduction of all trans retinal to retinol consumes 1 NADPH (= 3 ATP). This results in a budget of 600 × 3.3 = 1.98 × 10^3 ATP $cell^{-1}$ s^{-1}.

Total energy requirement for visual transduction at indoor light conditions: Summing up energies of steps 1 to 5 results in a total budget for visual transduction indoors of 1.05×10^6 ATP $cell^{-1}$ s^{-1}.

VISUAL TRANSDUCTION CONES

The main energy demand here is the guanylate cyclase, which is expressed at similar levels in cPR as in rPR. We therefore assumed similar energy demands in indoor light (1×10^6 ATP s^{-1} $cell^{-1}$).

VISUAL TRANSDUCTION RPE

Visual cycle RPE: Each RPE cell needs to take care of the retinal from 150000/ 4500 = 33 rod cells. The recycling part of retinal that occurs in the RPE requires 4 NADH[87] = 12 ATP. At indoor light conditions (33 x 600 Rh* s^{-1}) this corresponds to 33 × 12 × 600 = 2.38×10^5 ATP s^{-1} $cell^{-1}$.

References

1. R. Noske, F. Cornelius, R. J. Clarke, Investigation of the enzymatic activity of the Na+,K+-ATPase via isothermal titration microcalorimetry. Biochim Biophys Acta 1797, 1540-1545 (2010).

2. G. R. Corradi et al., Plasma Membrane Ca(2+) Pump PMCA4z Is More Active Than Splicing Variant PMCA4x. Front Cell Neurosci 15, 668371 (2021).

3. W. A. Hagins, R. D. Penn, S. Yoshikami, Dark current and photocurrent in retinal rods. Biophys J 10, 380-412 (1970).

4. D. J. Aidley, The physiology of excitable cells. (Cambridge University Press, Cambridge ; New York, ed. 2d, 1978), pp. ix, 530 p.

5. H. Okawa, A. P. Sampath, S. B. Laughlin, G. L. Fain, ATP consumption by mammalian rod photoreceptors in darkness and in light. Curr Biol 18, 1917-1921 (2008).

6. M. T. Wong-Riley, Energy metabolism of the visual system. Eye Brain 2, 99-116 (2010).

7. N. T. Ingram, G. L. Fain, A. P. Sampath, Elevated energy requirement of cone photoreceptors. Proc Natl Acad Sci U S A 117, 19599-19603 (2020).

8. J. W. Maddox et al., A dual role for Ca(v)1.4 Ca(2+) channels in the molecular and structural organization of the rod photoreceptor synapse. Elife 9, (2020).

9. D. Krizaj, D. R. Copenhagen, Compartmentalization of calcium extrusion mechanisms in the outer and inner segments of photoreceptors. Neuron 21, 249-256 (1998).

10. C. W. Morgans, O. El Far, A. Berntson, H. Wässle, W. R. Taylor, Calcium extrusion from mammalian photoreceptor terminals. J Neurosci 18, 2467-2474 (1998).

11. D. Cia et al., Voltage-gated channels and calcium homeostasis in mammalian rod photoreceptors. J Neurophysiol 93, 1468-1475 (2005).

12. R. W. Young, The renewal of rod and cone outer segments in the rhesus monkey. J Cell Biol 49, 303-318 (1971).

13. M. Dvoriashyna, A. J. E. Foss, E. A. Gaffney, R. Repetto, Fluid and solute transport across the retinal pigment epithelium: a theoretical model. J R Soc Interface 17, 20190735 (2020).

14. I. Ignatova, R. Frolov, S. Nymark, The retinal pigment epithelium displays electrical excitability and lateral signal spreading. BMC Biol 21, 84 (2023).

15. E. M. De La Cruz, E. M. Ostap, Kinetic and equilibrium analysis of the myosin ATPase. Methods Enzymol 455, 157-192 (2009).

16. T. Yanagida, A. H. Iwane, A large step for myosin. Proc Natl Acad Sci U S A 97, 9357-9359 (2000).

17. B. Spreng et al., Microtubule number and length determine cellular shape and function in Plasmodium. Embo j 38, e100984 (2019).

18. A. J. Zwetsloot, G. Tut, A. Straube, Measuring microtubule dynamics. Essays Biochem 62, 725-735 (2018).

19. J. C. Cochran, Kinesin Motor Enzymology: Chemistry, Structure, and Physics of Nanoscale Molecular Machines. Biophys Rev 7, 269-299 (2015).

20. D. L. Coy, M. Wagenbach, J. Howard, Kinesin takes one 8-nm step for each ATP that it hydrolyzes. J Biol Chem 274, 3667-3671 (1999).

21. F. Gibbons, J. F. Chauwin, M. Despósito, J. V. José, A dynamical model of kinesin-microtubule motility assays. Biophys J 80, 2515-2526 (2001).

22. S. Niekamp, N. Coudray, N. Zhang, R. D. Vale, G. Bhabha, Coupling of ATPase activity, microtubule binding, and mechanics in the dynein motor domain. Embo j 38, e101414 (2019).

23. A. B. Goryachev, A. V. Pokhilko, Computational model explains high activity and rapid cycling of Rho GTPases within protein complexes. PLoS Comput Biol 2, e172 (2006).

24. B. Schwanhäusser et al., Global quantification of mammalian gene expression control. Nature 473, 337-342 (2011).

25. L. Brocchieri, S. Karlin, Protein length in eukaryotic and prokaryotic proteomes. Nucleic Acids Res 33, 3390-3400 (2005).

26. B. S. Jo, S. S. Choi, Introns: The Functional Benefits of Introns in Genomes. Genomics Inform 13, 112-118 (2015).

27. R. W. Swick, Measurement of protein turnover in rat liver. J Biol Chem 231, 751-764 (1958).

28. W. Kwon, S. A. Freeman, Phagocytosis by the Retinal Pigment Epithelium: Recognition, Resolution, Recycling. Front Immunol 11, 604205 (2020).

29. C. Sun, E. M. Schuman, Logistics of neuronal protein turnover: Numbers and mechanisms. Mol Cell Neurosci 123, 103793 (2022).

30. C. A. Hirsch, H. H. Hiatt, Turnover of liver ribosomes in fed and in fasted rats. J Biol Chem 241, 5936-5940 (1966).

31. C. Jouffe et al., The circadian clock coordinates ribosome biogenesis. PLoS Biol 11, e1001455 (2013).

32. A. S. Stoykova, K. P. Dudov, M. D. Dabeva, A. A. Hadjiolov, Different rates of synthesis and turnover of ribosomal RNA in rat brain and liver. J Neurochem 41, 942-949 (1983).

33. P. Umate, N. Tuteja, R. Tuteja, Genome-wide comprehensive analysis of human helicases. Commun Integr Biol 4, 118-137 (2011).

34. C. P. Toseland, M. R. Webb, ATPase mechanism of the 5'-3' DNA helicase, RecD2: evidence for a pre-hydrolysis conformation change. J Biol Chem 288, 25183-25193 (2013).

35. M. C. Wahl, C. L. Will, R. Lührmann, The spliceosome: design principles of a dynamic RNP machine. Cell 136, 701-718 (2009).

36. J. Hnilicová, D. Staněk, Where splicing joins chromatin. Nucleus 2, 182-188 (2011).

37. J. Houseley, D. Tollervey, The many pathways of RNA degradation. Cell 136, 763-776 (2009).

38. D. Shore, B. Albert, Ribosome biogenesis and the cellular energy economy. Curr Biol 32, R611-r617 (2022).

39. D. Kressler, E. Hurt, J. Bassler, Driving ribosome assembly. Biochim Biophys Acta 1803, 673-683 (2010).

40. S. M. Plafker, I. G. Macara, Ribosomal protein L12 uses a distinct nuclear import pathway mediated by importin 11. Mol Cell Biol 22, 1266-1275 (2002).

41. R. Martin, A. U. Straub, C. Doebele, M. T. Bohnsack, DExD/H-box RNA helicases in ribosome biogenesis. RNA Biol 10, 4-18 (2013).

42. E. Chapman, G. W. Farr, W. A. Fenton, S. M. Johnson, A. L. Horwich, Requirement for binding multiple ATPs to convert a GroEL ring to the folding-active state. Proc Natl Acad Sci U S A 105, 19205-19210 (2008).

43. P. Vlastaridis et al., Estimating the total number of phosphoproteins and phosphorylation sites in eukaryotic proteomes. Gigascience 6, 1-11 (2017).

44. H. J. An, J. W. Froehlich, C. B. Lebrilla, Determination of glycosylation sites and site-specific heterogeneity in glycoproteins. Curr Opin Chem Biol 13, 421-426 (2009).

45. Q. Zhang, C. Ma, L. S. Chin, L. Li, Integrative glycoproteomics reveals protein N-glycosylation aberrations and glycoproteomic network alterations in Alzheimer's disease. Sci Adv 6, (2020).

46. A. Peth, J. A. Nathan, A. L. Goldberg, The ATP costs and time required to degrade ubiquitinated proteins by the 26 S proteasome. J Biol Chem 288, 29215-29222 (2013).

47. J. A. M. Bard, C. Bashore, K. C. Dong, A. Martin, The 26S Proteasome Utilizes a Kinetic Gateway to Prioritize Substrate Degradation. Cell 177, 286-298.e215 (2019).

48. J. J. Tomashek, W. S. Brusilow, Stoichiometry of energy coupling by proton-translocating ATPases: a history of variability. J Bioenerg Biomembr 32, 493-500 (2000).

49. H. Sze, X. Li, M. G. Palmgren, Energization of plant cell membranes by H+-pumping ATPases. Regulation and biosynthesis. Plant Cell 11, 677-690 (1999).

50. J. Zhao, S. Benlekbir, J. L. Rubinstein, Electron cryomicroscopy observation of rotational states in a eukaryotic V-ATPase. Nature 521, 241-245 (2015).

51. M. Nakanishi-Matsui, M. Sekiya, R. K. Nakamoto, M. Futai, The mechanism of rotating proton pumping ATPases. Biochim Biophys Acta 1797, 1343-1352 (2010).

52. M. E. Finbow, M. A. Harrison, The vacuolar H+-ATPase: a universal proton pump of eukaryotes. Biochem J 324 (Pt 3), 697-712 (1997).

53. H. Imamura et al., Evidence for rotation of V1-ATPase. Proc Natl Acad Sci U S A 100, 2312-2315 (2003).

54. S. Ramachandra Rao, S. J. Fliesler, Cholesterol homeostasis in the vertebrate retina: biology and pathobiology. J Lipid Res 62, 100057 (2021).

55. W. D. Blaker, A. D. Toews, P. Morell, Cholesterol is a component of the rapid phase of axonal transport. J Neurobiol 11, 243-250 (1980).

56. S. J. Fliesler, R. Florman, L. M. Rapp, S. J. Pittler, R. K. Keller, In vivo biosynthesis of cholesterol in the rat retina. FEBS Lett 335, 234-238 (1993).

57. R. K. Keller, S. J. Fliesler, Incorporation of squalene into rod outer segments. J Biol Chem 265, 13709-13712 (1990).

58. R. K. Keller, S. J. Fliesler, S. W. Nellis, Isoprenoid biosynthesis in the retina. Quantitation of the sterol and dolichol biosynthetic pathways. J Biol Chem 263, 2250-2254 (1988).

59. A. D. Albert, K. Boesze-Battaglia, The role of cholesterol in rod outer segment membranes. Prog Lipid Res 44, 99-124 (2005).

60. M. Prentki, S. R. Madiraju, Glycerolipid metabolism and signaling in health and disease. Endocr Rev 29, 647-676 (2008).

61. T. Omura, P. Siekevitz, G. E. Palade, Turnover of constituents of the endoplasmic reticulum membranes of rat hepatocytes. J Biol Chem 242, 2389-2396 (1967).

62. G. W. Hughes et al., MlaFEDB displays flippase activity to promote phospholipid transport towards the outer membrane of Gram-negative bacteria. bioRxiv, 2020.2006.2006.138008 (2020).

63. A. M. Landeira-Fernandez, A. Galina, L. de Meis, Catalytic activity and heat production by the Ca(2+)-ATPase from sea cucumber (Ludwigothurea grisea) longitudinal smooth muscle: modulation by monovalent cations. J Exp Biol 203, 3613-3619 (2000).

64. C. G. Fraga, M. K. Shigenaga, J. W. Park, P. Degan, B. N. Ames, Oxidative damage to DNA during aging: 8-hydroxy-2'-deoxyguanosine in rat organ DNA and urine. Proc Natl Acad Sci U S A 87, 4533-4537 (1990).

65. M. Yousefzadeh et al., DNA damage-how and why we age? Elife 10, (2021).

66. T. Lindahl, Instability and decay of the primary structure of DNA. Nature 362, 709-715 (1993).

67. J. Nakamura, J. A. Swenberg, Endogenous apurinic/apyrimidinic sites in genomic DNA of mammalian tissues. Cancer Res 59, 2522-2526 (1999).

68. M. M. Vilenchik, A. G. Knudson, Endogenous DNA double-strand breaks: production, fidelity of repair, and induction of cancer. Proc Natl Acad Sci U S A 100, 12871-12876 (2003).

69. S. P. Jackson, J. Bartek, The DNA-damage response in human biology and disease. Nature 461, 1071-1078 (2009).

70. J. P. Melis, H. van Steeg, M. Luijten, Oxidative DNA damage and nucleotide excision repair. Antioxid Redox Signal 18, 2409-2419 (2013).

71. A. N. Lane, T. W. Fan, Regulation of mammalian nucleotide metabolism and biosynthesis. Nucleic Acids Res 43, 2466-2485 (2015).

72. Y. Krasikova, N. Rechkunova, O. Lavrik, Nucleotide Excision Repair: From Molecular Defects to Neurological Abnormalities. Int J Mol Sci 22, (2021).

73. N. Riveros, J. Fiedler, N. Lagos, C. Muñoz, F. Orrego, Glutamate in rat brain cortex synaptic vesicles: influence of the vesicle isolation procedure. Brain Res 386, 405-408 (1986).

74. L. M. Levy, O. Warr, D. Attwell, Stoichiometry of the glial glutamate transporter GLT-1 expressed inducibly in a Chinese hamster ovary cell line selected for low endogenous Na+-dependent glutamate uptake. J Neurosci 18, 9620-9628 (1998).

75. H. Wolosker, D. O. de Souza, L. de Meis, Regulation of glutamate transport into synaptic vesicles by chloride and proton gradient. J Biol Chem 271, 11726-11731 (1996).

76. D. Attwell, S. B. Laughlin, An energy budget for signaling in the grey matter of the brain. J Cereb Blood Flow Metab 21, 1133-1145 (2001).

77. Z. Sheng et al., Synaptic Ca2+ in darkness is lower in rods than cones, causing slower tonic release of vesicles. J Neurosci 27, 5033-5042 (2007).

78. S. L. Jackman et al., Role of the synaptic ribbon in transmitting the cone light response. Nat Neurosci 12, 303-310 (2009).

79. D. Fotiadis et al., Atomic-force microscopy: Rhodopsin dimers in native disc membranes. Nature 421, 127-128 (2003).

80. A. Kelber, Vision: Rods See in Bright Light. Curr Biol 28, R364-r366 (2018).

81. T. D. Lamb, T. W. Kraft, A quantitative account of mammalian rod phototransduction with PDE6 dimeric activation: responses to bright flashes. Open Biol 10, 190241 (2020).

82. T. D. Lamb, Photoreceptor physiology and evolution: cellular and molecular basis of rod and cone phototransduction. J Physiol 600, 4585-4601 (2022).

83. B. M. Qureshi et al., It takes two transducins to activate the cGMP-phosphodiesterase 6 in retinal rods. Open Biol 8, (2018).

84. M. Heck, K. P. Hofmann, T. W. Kraft, T. D. Lamb, Phototransduction gain at the G-protein, transducin, and effector protein, phosphodiesterase-6, stages in retinal rods. Proc Natl Acad Sci U S A 116, 8653-8654 (2019).

85. J. B. Hurley, M. Spencer, G. A. Niemi, Rhodopsin phosphorylation and its role in photoreceptor function. Vision Res 38, 1341-1352 (1998).

86. S. C. Hsu, R. S. Molday, Glucose metabolism in photoreceptor outer segments. Its role in phototransduction and in NADPH-requiring reactions. J Biol Chem 269, 17954-17959 (1994).

87. P. D. Kiser, M. Golczak, A. Maeda, K. Palczewski, Key enzymes of the retinoid (visual) cycle in vertebrate retina. Biochim Biophys Acta 1821, 137-151 (2012).

Supplementary Figures

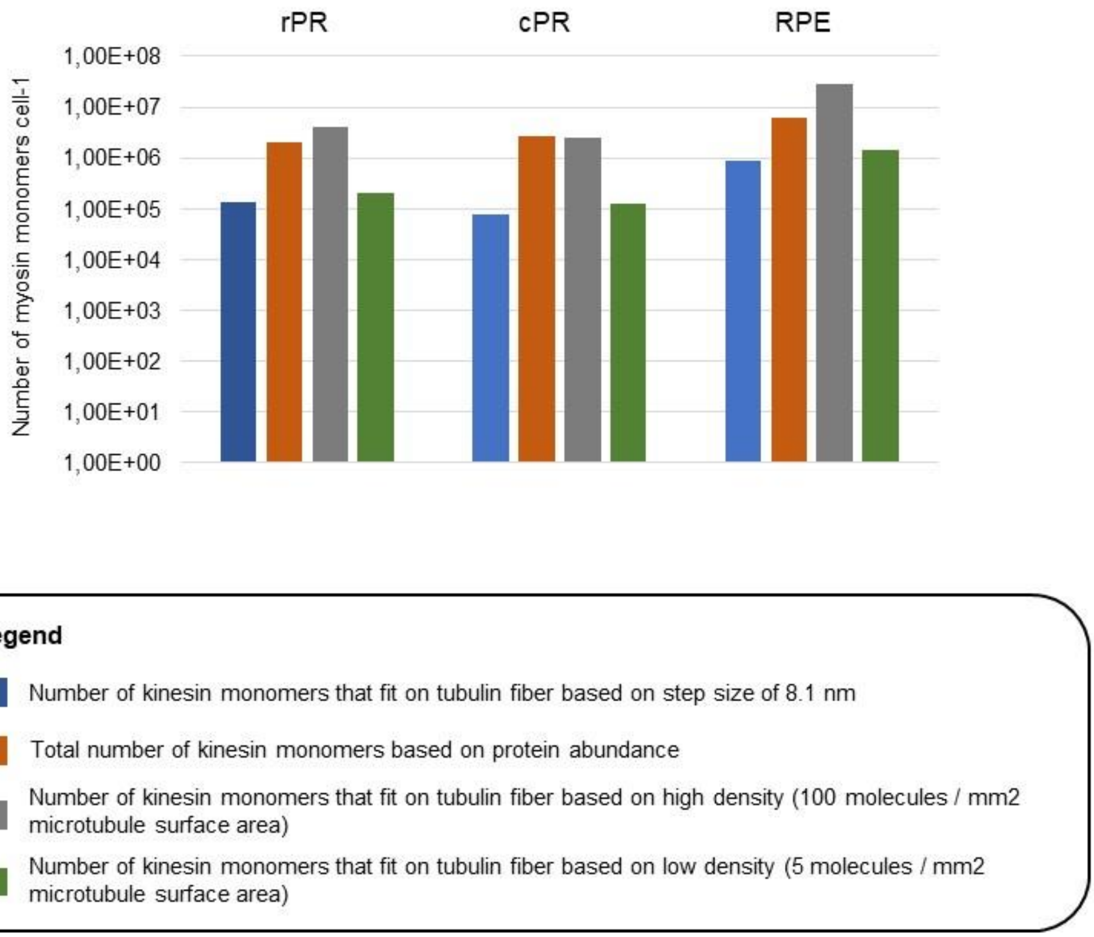

Supplementary Fig. S1. Comparison of free and microtubule-bound kinesin molecules estimated based on protein abundances, the total length and area of microtubules, and based on different kinesin densities. Calculations are shown for rPR cells.

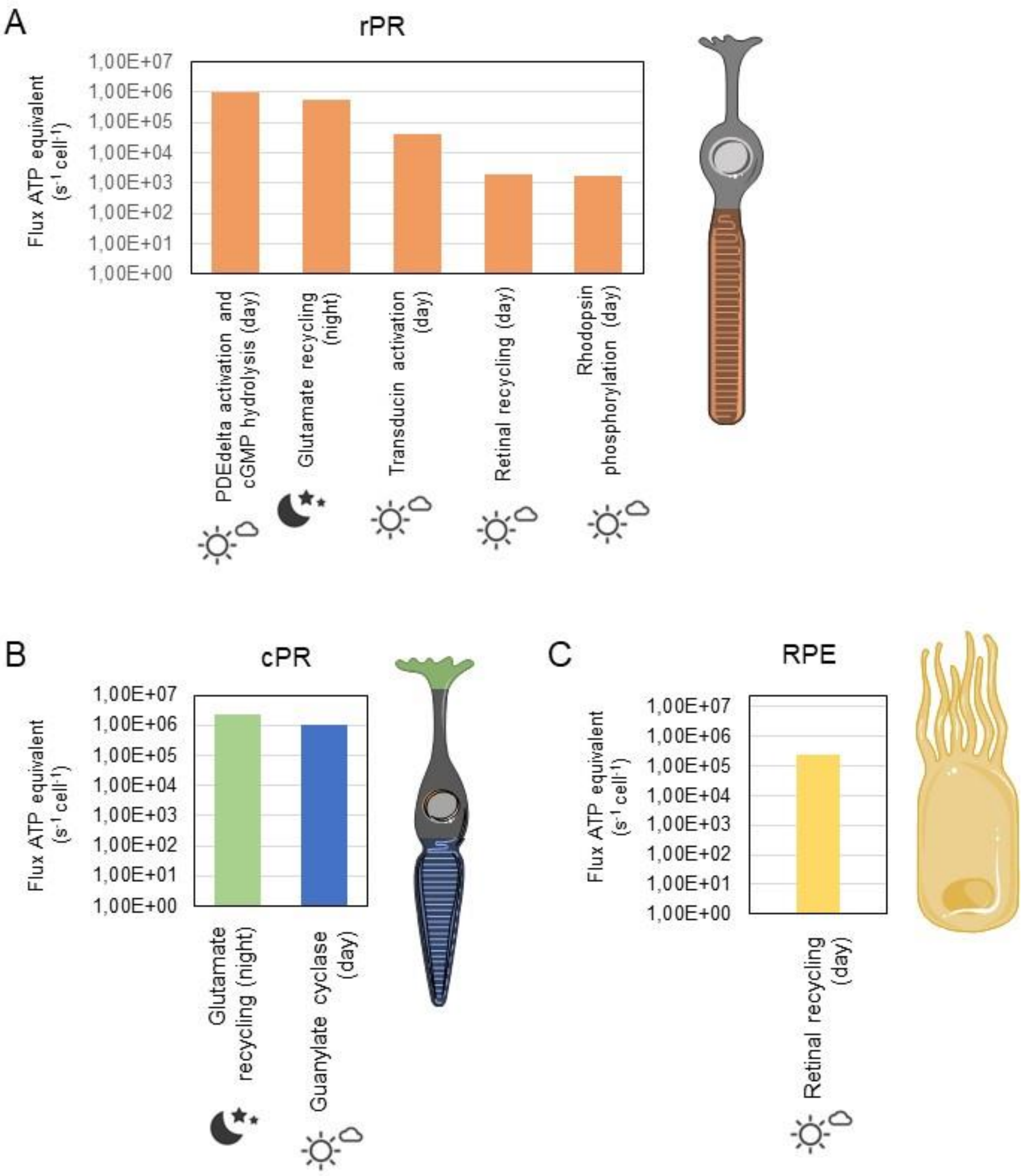

Supplementary Fig. S2. Energy demands for outer retina specific processes. (**A**) ATP fluxes per cell in rPR cells for visual transduction (day) and glutamate recycling (night). Note that the dark current is covered under PM ion transport. (**B**) ATP fluxes per cell in cPR cells for glutamate recycling at night and guanylate cyclase during the day. (**C**) ATP fluxes per cells in RPE cells for retinal recycling (day).

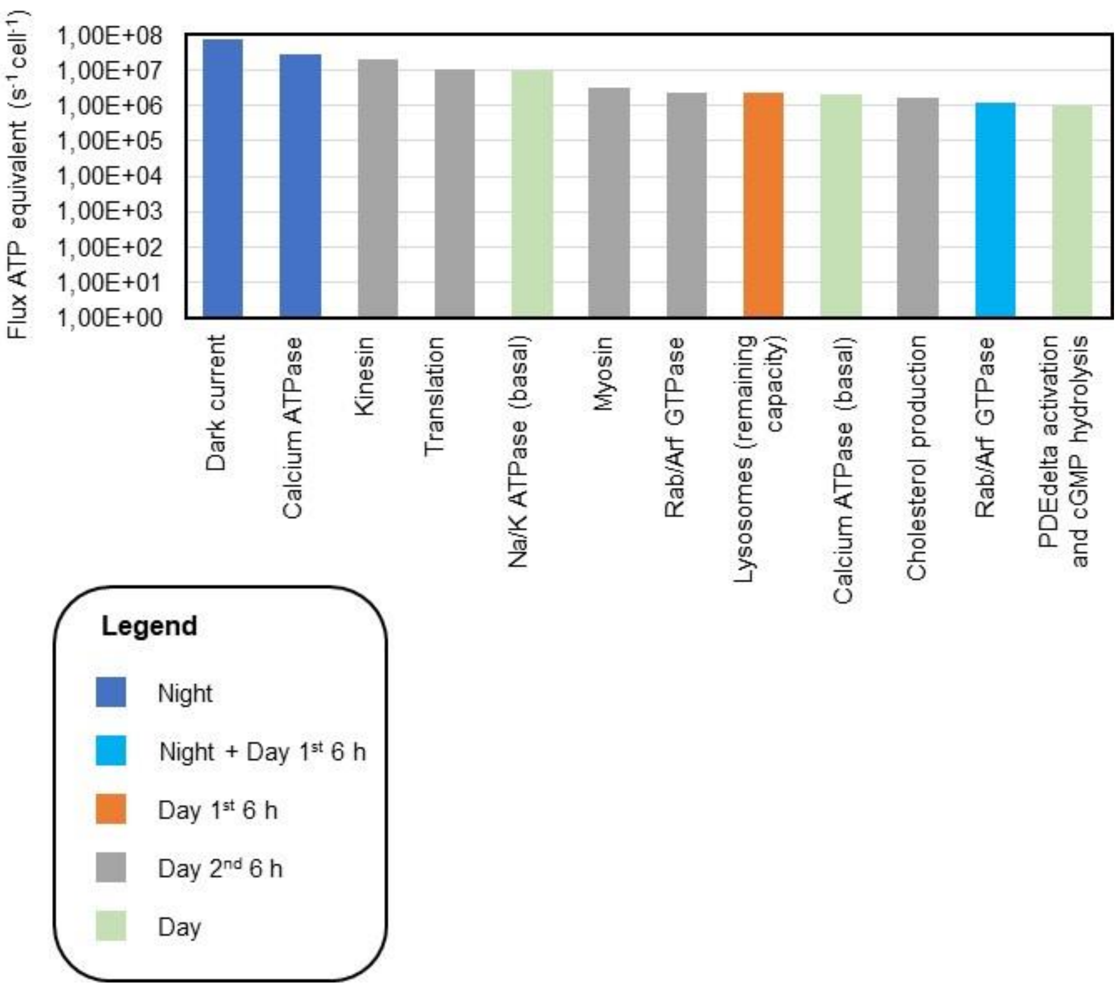

Supplementary Fig. S3. Processes with highest (> 1 x 10^6 s^{-1}) ATP fluxes per cell for rPR cells. The bar graphs of fluxes are coloured according to the time frame during which the process is predicted to be active (see legend).

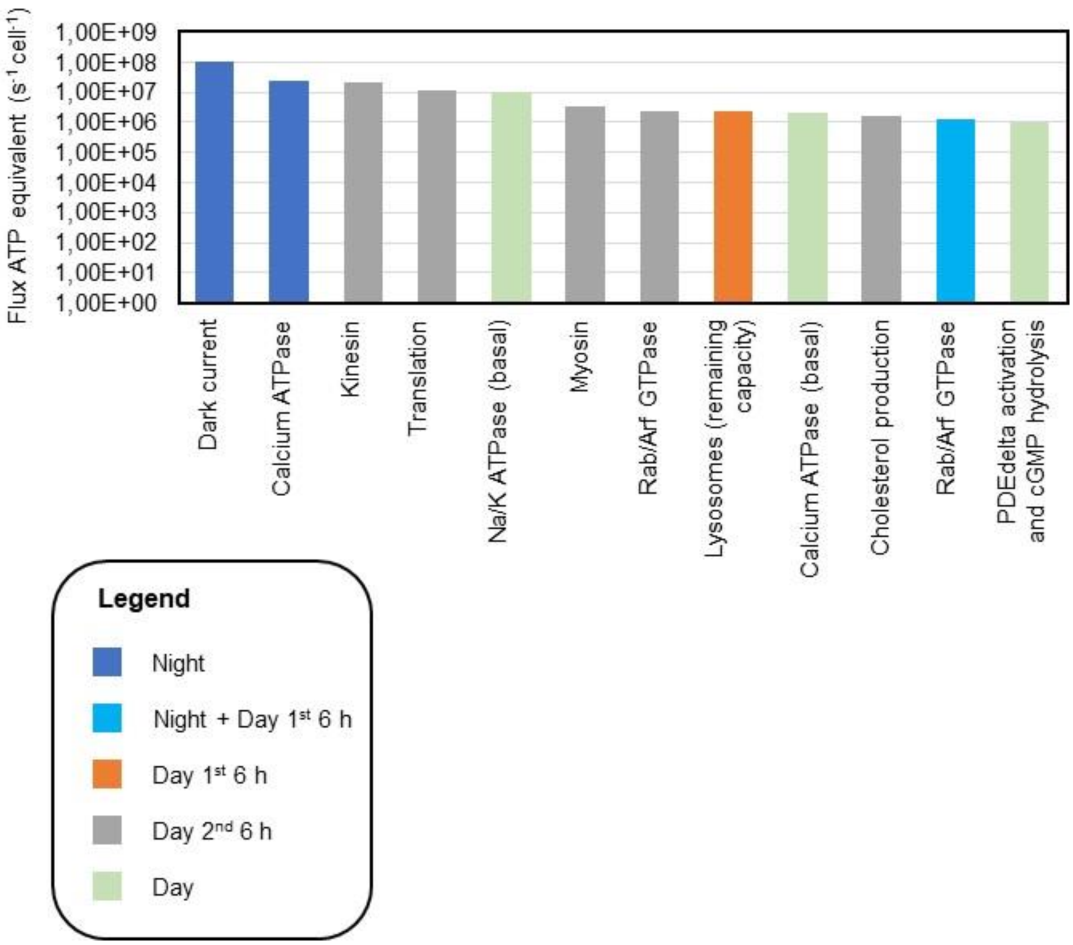

Supplementary Fig. S4. Processes with highest (> 1 x 10^6 s^{-1}) ATP fluxes per cell for cPR cells. The bar graphs of fluxes are coloured according to the time frame during which the process is predicted to be active (see legend).

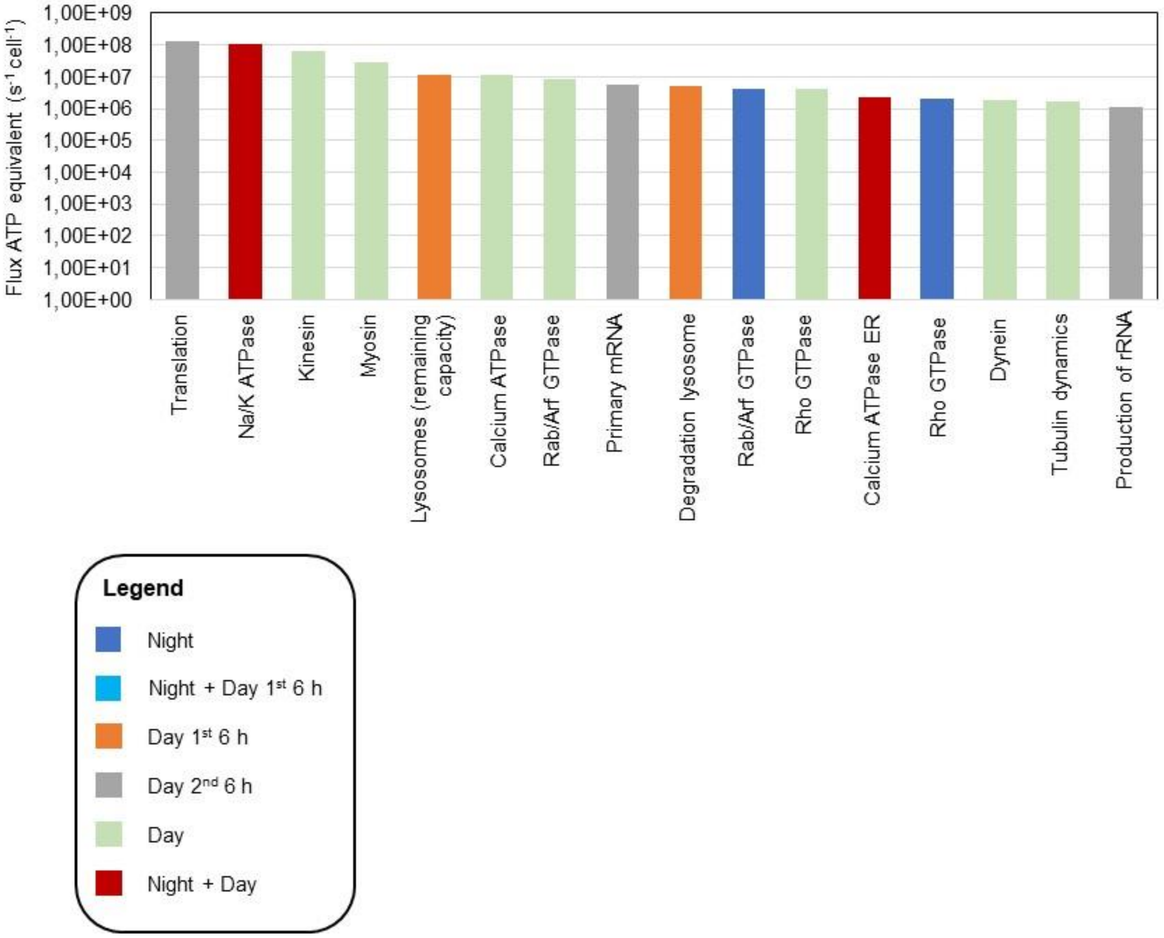

Supplementary Fig. S5. Processes with highest (> 1 x 10^6 s^{-1}) ATP fluxes per cell for RPE cells. The bar graphs of fluxes are coloured according to the time frame during which the process is predicted to be active (see legend).
