# Supplementary material for: “Energetics of the outer retina II: Calculation of a spatio-temporal energy budget in retinal pigment epithelium and photoreceptor cells based on quantification of cellular processes”
Source: PLoS One. 2025 Jan 27;20(1):e0311169. doi: 10.1371/journal.pone.0311169 (PMC11771881; doi:10.1371/journal.pone.0311169)
Supplement: S3 Table — (DOCX) [file pone.0311169.s004.docx]

**Supplementary Table S3.** Currency conversions.

| **Currency** | **ATP needed for conversion** | **ATP equivalent** |
| --- | --- | --- |
| ATP hydrolysis to AMP | 1 additional ATP is needed to regenerate ADP from AMP (phosphorylation of AMP by adenylate kinase) | 2 |
| GTP hydrolysis to GDP | 1 ATP is required to regenerate GTP from GDP (phosphorylation by nucleoside diphosphate kinase) | 1 |
| GTP hydrolysis to GMP | 2 ATP are required as GMP needs to be converted to GDP first (phosphorylation by guanylate cyclase) | 2 |
| UTP hydrolysis to UMP | 2 ATP are needed as UMP is phosphorylated to UDP (by nucleoside monophosphate kinase) and UDP is phosphorylated to UTP (by nucleoside diphosphokinase) | 2 |
| CTP hydrolysis to CMP | 2 ATP are required as CMP needs to be converted to CDP (phosphorylation by cytidylate kinase). CTP is generated from UTP using 1 ATP (by CTP synthase). | 2 |
| NADH (cytosol) |  | 2 |
| NADH (matrix) |  | 3 |
| FADH_2_ |  | 2 |
| NAD+ | NAD+ is resynthesized at a cost of 2 to 4 ATP molecules (Hoeijmakers, 2009) | 3 |
| Acetyl-CoA | Each Acetyl-CoA enters the TCA cycle and yields 3 NADH (+H+) (= 9 ATP), 1 FADH2 (= 2 ATP), and 1 GTP (= 1 ATP). Altogether 12 ATP can be produced. | 12 |
| Glucose via glycolysis to pyruvate | 1 glucose + 2 ATP + 2 NAD+ + 2 Pi -> 2 pyruvate + 2 NADH (cytosolic) + 2 H+ + 4 ATP + 2 H_2_0 | 7 |
| Pyruvate (cytosol) to Acetyl-CoA (mitochondrion) | 1 pyruvate + NAD+ + CoA -> Acetyl-CoA + NADH + H+ + CO2 | 15 |
| Glucose via glycolysis and oxidative phosphorylation | 1 glucose produces 2 ATP (glycolysis) + 2 NADH (cytosol via glycolysis) (= 4 ATP) + 2 NADH (mitochondrion from pyruvate) (= 6 ATP) + 2 ATP (TCA cycle) + 6 NADH (mitochondrion via TCA cycle) (= 18 ATP) + 2 FADH2 (mitochondrion via TCA cycle) (= 4 ATP). This results in a maximum of 36 ATP. Because of proton leakage, we assume here that 32 ATP are generated from 1 glucose molecule. | 32 |
