# Supplementary material for: “Energetics of the outer retina II: Calculation of a spatio-temporal energy budget in retinal pigment epithelium and photoreceptor cells based on quantification of cellular processes”
Source: PLoS One. 2025 Jan 27;20(1):e0311169. doi: 10.1371/journal.pone.0311169 (PMC11771881; doi:10.1371/journal.pone.0311169)
Supplement: Attachment — Submitted filename: 2024_PlosOne_Review of Kiel et al.pdf [file pone.0311169.s006.pdf]

2024 / 07 / 03

Review of: "Energetics of the outer retina II: Calculation of a spatio-temporal energy budget in retinal pigment epithelium and photoreceptor cells based on quantification of cellular processes"

This is a wonderful effort and is of value to the scientific community. Too few investigators work to integrate information from many labs into a coherent analysis, so I very much appreciate efforts such as this one.

While I believe that a future form of this is worth publishing, have quibbles about some of the details in the manuscript's current state. These are mostly geared towards using language that emphasizes the limitations of the manuscript to a greater extent. Some of the limitations are also due to specific assumptions that are difficult to believe would hold up realistically. These are noted.

(1) By using turnover number (K_{cat}) and a transcriptome-based assessment of enzyme levels (**line 92**), two major assumptions are made.
   a. The transcriptome and proteome correspond well to each other. This is not usually the case (PMIDs: 28438116**,** 20023718**,** 19850042). The concept that calculations of protein abundance or distribution could be incorrect is not discussed and should be, since this data is foundational to the rest of the study.
   b. A K_{cat} is determined for saturating concentration of the enzyme's substrate (here, often ATP). It seems unlikely that all enzymes simultaneously operate at maximum flux. This is a major assumption that would be questioned by many people if stated this way. Overall I do not think this study quantifies normal cellular energy budgets per se, rather it calculates maximum possible energy demands. The assumption that all ATPases would need to work at their K_{cat} is mentioned but not as a major limitation in the discussion. It should be. Additionally I recommend but will not require that the authors refer to their calculations as "maximums" calculated for a given cellular process, so that readers will not accidentally interpret the maximum possible ATP expenditures as physiological ATP expenditures. An example of this would be to transform the statement "we have quantified ATP (or ATP equivalent) fluxes" → ""we have calculated maximum possible ATP (or ATP equivalent) fluxes". I think this language would make the meaning of this data more clear to readers.

(2) **Line 67-69**: Are these lengths and widths of inner and outer segments arbitrary or derived from a specific source in the literature? Regardless of where it originated from, it would be best to refer to the source of information used for cell compartment volume calculations.

(3) **Line 141**: So many of the downstream measurements depend on cell volume. That cone photoreceptors are assumed to be the same volume as rods with no more evidence than they are shorter and also wider seems ridiculous and calls into question the analytical rigor of a paper that is incredibly reliant on a solid analytical approach to data analysis. The authors should cite evidence for their determination of cone volume or provide an analysis of how estimated cell volume impacts energy utilization measurements.

(4) **Line 143**: There are differences in RPE surface area and presumably volume as a function of retinal eccentricity. This means that in rod-rich areas of the retina vs. cone-rich areas, there will be distinct RPE cell volumes, which could throw off rod-RPE-ratio estimates. This should be mentioned as a confounding factor in the discussion.

(5) **Line 278**: The citation used to rationalize a 3-day protein half-life in RPE appears to be geared towards the liver. RPE and liver are of course different tissues with different needs, as are RPE and photoreceptors. This is one example of where values from a different tissue are substituted for values of a similar process in photoreceptors or RPE. The practice seems necessary for this manuscript in the absence of better data, but a statement in the discussion should note that values for the rate of a process from distinct tissues and species may not be true when estimating the activity of that process for the human eye.

(6) **Line 453**: Do the authors mean “OR” or “IS” instead of “OS”?

(7) **Line 505:** The last word in the sentence should be “anabolism”

(8) **Line 517**: No year on this citation

(9) There is no propagation of experimental error from original sources of data. In an ideal world, estimates of ATP expenditure by each cellular process would have error bars representing uncertainty that was propagated from the original papers. I realize this last point is perhaps too much to ask so this modification is an optional request that would enhance the manuscript but will not be required from me for publication.

(10) To an extent the authors change the K_{cat} for different circadian periods. These changes often appear to be arbitrary and values are not always linked to a citation. All K_{cat} values should either be cited in text, or a citation should be linked to the

supplemental spreadsheets. As the study stands some of the values seem to be cited but not all of them.

(11) Improvements in technology means that better retina, photoreceptor, or RPE-specific estimates of turnovers, PTMs, cytoskeletal movements, etc. might be published in the future. This study provides a framework for estimating energy expenditures that should ideally be updated as improved values for each process are determined experimentally. It is certainly not a requirement for the manuscript and is far outside my purview as a reviewer, but I highly recommend the authors consider generating a publicly available resource that uses the framework behind this study but can be updated. A web-based tool where people can deposit recommended values for a given process might be best. Such a strategy could drastically improve the usefulness and long-term accuracy of the current study.

Supplementary document:

- There are several instances in this section where assumptions are stated but the reasons for a given assumption are unstated. This leads to the reader (me) having the uneasy feeling that a substantial proportion of the calculations are determined using arbitrary starting values. In some cases the choice of constants could inform major outputs in this paper. Where possible, could the authors improve their explanations for a given assumption? This issue was noticeable in the following sections of the supplementary information: "We also assumed a higher k_{cat} (average of 0.5 and 80 s -1 = 40.25 s-1)", "We initially assumed that 50 % of the proteins are degraded via the proteasome and 50 % via the lysosome", ""
- The reference section has been added twice to this document
- For some information (for example the energetics costs from using Ca^{2+} ATPases), there is a range of possible ATP consumption values based on estimates and on published data. Yet in figures a single value is chosen to represent that energetic cost. Would it be feasible to represent ATP costs as a range of possible costs, where applicable? And not just for this section but wherever there is a known range of uncertain values in the calculation?
- The calculation of energetic costs associated with PTMs appear to be particularly unrealistic for a few reasons: (**1**) they assume every site on a protein that can be modified with a PTM is normally modified. It is normal in literature to see changes in the abundance of various PTM under different conditions, which indicates that not all possible PTM sites are normally occupied. This study would be improved by the incorporation of a term which quantifies the proportion of PTM sites that are normally occupied. (**2**) I think the ATP-equivalent values for production of molecules like acetyl-CoA are calculated incorrectly. The values used actually correspond with the energy that would be produced if acetyl-CoA were consumed. Use of as a PTM does

not use up the energy it does not generate. The calculation should instead have the cost of Acetyl-CoA synthesis. Acetyl-CoA synthesis shares most if its reactions with glycolysis, but should additionally reduce a molecule of NAD+ to NADH. This of course assumes there is no limitation on CoA availability. When the production of a molecule used to modify a protein is energetically productive it should be subtracted from the energetic budget rather than added to it. (**3**) Acetylation, glycosylation, phosphorylation, and ubiquitination are far from the only posttranslational modifications. If the authors plan to only determine the energy budget associated with only these modifications, they should provide a reasonable explanation for why the other modifications are not taken into account. Perhaps other modifications are far less abundant on proteins? If so, please find and cite this information.

- **Page 19**: Estimates of protein turnover may be slightly off. This paper (PMID: 7452249) is an estimate of protein synthesis for the whole rabbit retina, but average protein synthesis rates in photoreceptors are expected to be faster than for other retinal cells. If I've calculated correctly from this paper, the estimate for the average retina protein half-life is almost exactly 4 days. Please consider using retina-specific estimates of turnover where possible.
- **Page 21:** I think "24 night and dark cycles" is meant to mean "24-hour light and dark cycle"
