# Supplementary material for: “Energetics of the outer retina II: Calculation of a spatio-temporal energy budget in retinal pigment epithelium and photoreceptor cells based on quantification of cellular processes”
Source: PLoS One. 2025 Jan 27;20(1):e0311169. doi: 10.1371/journal.pone.0311169 (PMC11771881; doi:10.1371/journal.pone.0311169)
Supplement: Attachment — Submitted filename: Reply to reviewers FINAL.docx [file pone.0311169.s007.docx]

Reviewer #1: Review for PONE-D-24-23593 - Energetics of the outer retina II: Calculation of a spatio-temporal energy budget in retinal pigment epithelium and photoreceptor cells based on quantification of cellular processes, Kiel et al., 2024

“*The manuscript by Kiel and colleagues tries to give detailed estimates of the energy consumption and the related cellular processes for the key cell types of the outer retina, namely retinal pigment epithelial (RPE) cells, and rod and cone photoreceptors. They combine gene expression data with biochemical knowledge to infer the ATP expenditure required for all major cellular processes and thereby obtain an unprecedented overview of outer retina energy consumption. Notably also because of its relevance for retinal disease pathogenesis, the topic of this article is highly attractive and appears well suited for PLoS One.
The manuscript is well written and the rather complex topic at hand is easy to follow. The figures shown generally are of high quality, with only minor shortcomings. However, some parts of the results may have been oversimplified and the authors appear to have missed the key role of Na+/K+/Ca2+ exchanger (NCKX) in regulating photoreceptor Ca2+ levels. These and other (mostly minor) issues must be appropriately addressed in a revised manuscript (see below).”*

*Major points:*
Comment 1: “*1. Results: The authors mention the key importance of quantitative data for their calculations and they employ gene expression data for their estimates of protein expression. This is problematic as gene expression and protein expression may not correlate well with each other, especially in low abundance transcripts. Here, even minor differences could lead to major distortions. For instance, the abundance of NKX inferred from gene expression could be an underestimation. While in the absence of quantitative single-cell proteomics there is no real solution to this problem, the problem as such and the possible misinterpretations (or limits to the current interpretation) should be mentioned clearly and appropriately discussed.*”

Response 1: We thank the reviewer for bringing up this important problem. We have added now a paragraph in the discussion to emphasize limitations arising from this problem.

Comment 2: “*2. Results:* *In several instances the assumptions and calculations appear to have been oversimplified or are not properly presented. For example, the authors state that rods and cones “have an inward Ca2+ current in the dark” (line 196) without saying what the current is that they used for their subsequent ATP-flux calculations. This is similar for most parts of the results (e.g. for Na+ influx, actin fiber polymerization and depolymerization, etc.) where the key values and assumptions that were used to make calculations should always be given in the main text.*”

Response 2: Because of the many assumptions used that we detail in the Supplemental text, we had indeed omitted information in the main text. We agree that we have omitted too much of the critical information from the main text. We now provide the critical information also in the main text, particularly for cytoskeleton-related processes.

Comment 3: “*3. Results: In the first part the authors suggest that ATP consumption from NKX activity was on par with or even lower than that from PMCA activity. This is in contradiction to earlier works that found NKX to consume at least 50% of the total photoreceptor ATP production (e.g. Ames et al., J Neurosci 12:840-853, 1992) and is somewhat counterintuitive given that the CNG-channel allows mostly for Na+ influx (only about 15-20% of the CNG current is carried by Ca2+).
Importantly, the authors seem to have overlooked that most of the Ca2+ influx mediated by the CNG-channel is compensated for by the Na+-gradient driven Na+/K+/Ca2+ exchanger (NCKX) in the outer segment (exchange of 1x Ca2+ and 1x K+ for 4x Na+). Hence, overall the Na+ influx and K+ outflux should be dramatically higher than the net Ca2+ influx, and, correspondingly, ATP-consumption for Ca2+ extrusion would be expected to be at least 1 order of magnitude lower than that for (re)establishing Na+/K+ gradients (through NKX activity).
The importance of NCKX for Ca2+ in the outer segment is also mirrored by the distribution of plasma membrane Ca2+-ATPase (PMCA) mostly to the synapse (something that the authors mention in the discussion). Without NCKX, PMCA would need to be far more strongly expressed in the inner segment to prevent Ca2+ from interfering with protein translation and gene expression.
The role of NCKX for photoreceptor Ca2+ levels must be acknowledged in the manuscript and the corresponding calculations should be revised.*”

Response 3: We appreciate that the reviewer spotted this. We have indeed made a mistake in estimating of ATP required for Na+/K+ and plasma membrane (PM) Ca2+ ATPase in the dark. We have corrected this in the revised version in the main text, figures and supplements.

In short, our estimates and assumptions are summarized for the reviewer below:

-the dark current is 40 pA per rod cell

-85% of the dark current is taken by Na+ and 15% Ca2+. This is 34 pA for Na+ and 6 pA for Ca2+

-the 6 pA Ca2+ current are removed via Ca2+-Na+-exchanger (NCKX) (4 Na+ in for 1 Ca2+ out) (that incoming Na+ is then handled by the Na+/K+ ATPase, NKX)

-the 6 pA that are handled by the Na+/K+ ATPase must be adjusted (multiplied by 2) as the charge ratio is 4 to 2 (4 Na+ for 1 Ca2+). This results in 12 pA.

-the total current that is handled by the Na+/K+ ATPase is therefore 34 pA + 12 pA = 46 pA

-there is a potassium leakage of 7% of the dark current. Therefore, we need to increase 46 pA by 7%, which is 49.22 pA.

-as 3 Na+ are pumped for one ATP, the current must be divided by 3 = 16.41 pA = 16.41 pC s^-1^ = 16.41 pC s^-1^ / 96485 C mol^-1^ = 0.00017 pmol s^-1^ = 1.02 x 10^8^ ATP molecules s^-1^ rod^-1^

There is an additional Ca2+ current in the dark of 4 pA, which is removed via PM Ca2+ ATPases in IS, cell body and synapse.

-conversion of 4 pA into ATP flux for PM Ca2+-ATPase (using similar conversions and 1 ATP per 1 Ca2+): 2.5 x 10^7^ ATP molecules s^-1^ rod^-1^

-in summary, we have now 1.02 x 10^8^ ATP s^-1^ rod^-1^for Na+/K+ ATPase and 2.50 x 10^7^ ATP s^-1^ rod^-1^ for PM Ca2+-ATPase (before the values were 6.97 10^7^ and 3 x 10^7^ ATP molecules s^-1^ rod^-1^)

We have updated this in the main text, supplemental information and figures.

Comment 4: “*4. All figures: Why are there no error bars in the bar graphs? The various measurements/estimates surely will have errors (or upper and lower boundaries) attached to them, these should also be included of the visualization of ATP fluxes.*”

Response 4: We have decided not to display errors bars in the visualization of ATP consumption diagrams as the ATP fluxes are not the result of one experimental uncertainty. The error associated with the ATP fluxes will be a combination of errors associated with experimental measurements (that have a quantifiable uncertainty) and assumptions that we take (that do not have a quantifiable uncertainty). [Note, that our ATP consumption rates are represented similar as previous energy budgets on the brain (PMID: 11598490) and in rod and cone PR (PMID: 19084410; PMID: 32719136) that also do not include error bars.]

However, we agree with the reviewer that these (different types of) uncertainties should be communicated to the readers. Therefore, we added a paragraph at the end of the methods section to explain (and quantify, where possible) the sources of uncertainties and errors. Particularly, we highlighted processes that include many assumptions that we took and that hence might be associated with larger uncertainties.

Comment 5: “*5. Figure 1b: In my version of the manuscript, the labels of the bar graphs are cut off and only partly visible.*”

Response 5: We have corrected this.

Comment 6: “*6.* *Figure 5:* *The pie charts should include numerical values for the different pie parts.*”
Response 6: We have done this.

Comment 7: “*7.* *Figure 6: The authors should add a scaling to the x-axis, and perhaps vertical dashed lines, to more clearly indicate what the energy expenditure of the different tissues is.*”
Response 7: We have done this.

Minor points
Comment 8: “*1. Abstract: The following sentence should probably be revised: “We propose the likely need of for diurnal…”.*”

Response 8: We have corrected this.

Comment 9: “*2. Line 109: double plural: “…copies for each proteins was…”*”

Response 9: We have corrected this.

Comment 10: “*3. The scaling of ATP flux in the various figures is variable, going from 1.00E+00 to 1.00E+07 to 1.00E+09. To allow an easier comparison across figures it would be helpful if the same logarithmic scale was used throughout all figures.*”

Response 10: We have scaled all bar diagrams in the same way now.

Reviewer #2:  “*This is a wonderful effort and is of value to the scientific community. Too few investigators work to integrate information from many labs into a coherent analysis, so I very much appreciate efforts such as this one.
While I believe that a future form of this is worth publishing, have quibbles about some of the details in the manuscript’s current state. These are mostly geared towards using language that emphasizes the limitations of the manuscript to a greater extent. Some of the limitations are also due to specific assumptions that are difficult to believe would hold up realistically. These are noted with other comments below.*”

Comment 11: “*(1) By using turnover number (Kcat) and a transcriptome-based assessment of enzyme levels (line 92), two major assumptions are made:
a. The transcriptome and proteome correspond well to each other. This is not usually the case (PMIDs: 28438116, 20023718, 19850042). The concept that calculations of protein abundance or distribution could be incorrect is not discussed and should be, since this data is foundational to the rest of the study.
b. A Kcat is determined for saturating concentration of the enzyme’s substrate (here, often ATP). It seems unlikely that all enzymes simultaneously operate at maximum flux. This is a major assumption that would be questioned by many people if stated this way. Overall I do not think this study quantifies normal cellular energy budgets per se, rather it calculates maximum possible energy demands. The assumption that all ATPases would need to work at their Kcat is mentioned but not as a major limitation in the discussion. It should be. Additionally I recommend but will not require that the authors refer to their calculations as “maximums” calculated for a given cellular process, so that readers will not accidentally interpret the maximum possible ATP expenditures as physiological ATP expenditures. An example of this would be to transform the statement “we have quantified ATP (or ATP equivalent) fluxes” ◊ ““we have calculated maximum possible ATP (or ATP equivalent) fluxes”. I think this language would make the meaning of this data more clear to readers.*”

Response 11: We thank the reviewer for bringing these two important assumptions up. Regarding point a (correlation of protein and mRNA abundance), we have added now a paragraph in the discussing to emphasize limitations arising from this problem.

Regarding point b (ATP fluxes estimated from kcat and enzyme abundance), the reviewer is right that that in many cases those ATP fluxes will represent upper limits. However, not in all cases. First, not all processes were calculated based on enzyme abundancies and kcat. For example, processes in the different categories of mRNA, ribosome, and protein turnover were based on “demand” (how many biomolecules turn over and must be replaced in a given time). Likewise, transport of ions (dark current) and also processes linked to visual transduction were estimated based on demand. Second, for some processes that were calculated based on enzyme abundances and kcat, there were additional assumptions with respect to time period of frequency of a particular process. This applies specifically to motor proteins moving on actin or tubulin fibres. In those cases the ATP fluxes are also not upper limits, as occupancy or speed of moving of actin/tubulin may have been underestimated.

What we did in the manuscript is to emphasise in which cases the ATP fluxes are likely upper limits.

Comment 12: “*(2) Line 67-69: Are these lengths and widths of inner and outer segments arbitrary or derived from a specific source in the literature? Regardless of where it originated from, it would be best to refer to the source of information used for cell compartment volume calculations.*”

Response 12: Yes, these quantities were obtained from the literature. We now provide the detailed information for the estimates (see also reply to comment 13)

However, we want to emphasise here that the actual sizes do not matter too much as although the energy budget is generated for a cell with a specific volume, the budget would scale linearly with cell volume. This is because the total number of proteins in a cell is scaled to cell volume. So, the reader could easily scale the energy consumption terms to a new cell volume. We mention this now in the method section.

Comment 13: “*(3) Line 141: So many of the downstream measurements depend on cell volume. That cone photoreceptors are assumed to be the same volume as rods with no more evidence than they are shorter and also wider seems ridiculous and calls into question the analytical rigor of a paper that is incredibly reliant on a solid analytical approach to data analysis. The authors should cite evidence for their determination of cone volume or provide an analysis of how estimated cell volume impacts energy utilization measurements.*”

Response 13: The reviewer is totally correct. We now updated the method section, where we detail the simple estimates for the volumes (including references) used for the energy budget. In particular we emphasise that we are focussing on foveal cones and that cell size and relative cell sub-compartment sizes vary greatly with eccentricity. We also mention that energy usage is assumed to linearly scale with cell volume. This is because we assume that protein abundance scales with cell volume. Hence, readers could easily adapt the energy budget to other cell volume. We mention that cell volumes can vary depending on the region of the retina.

Comment 14: “*(4) Line 143: There are differences in RPE surface area and presumably volume as a function of retinal eccentricity. This means that in rod-rich areas of the retina vs. cone-rich areas, there will be distinct RPE cell volumes, which could throw off rod-RPE-ratio estimates. This should be mentioned as a confounding factor in the discussion.*”

Response 14: This relates also to the replies to comments 12 and 13. Here, the reviewer is right on the level of tissue area, the estimates for numbers of all cell types (and volumes) need to be absolutely correct for correct per area energy demand predictions. To be frank with the reviewer, the authors had endless discussions about the “correct” size of the different cell types. It is not an issue that can be solved in a systematic way. Particularly the number/volume per mm^2^ issue.

We emphasize this as another limitation in the discussion.

Comment 15: “*(5) Line 278: The citation used to rationalize a 3-day protein half-life in RPE appears to be geared towards the liver. RPE and liver are of course different tissues with different needs, as are RPE and photoreceptors. This is one example of where values from a different tissue are substituted for values of a similar process in photoreceptors or RPE. The practice seems necessary for this manuscript in the absence of better data, but a statement in the discussion should note that values for the rate of a process from distinct tissues and species may not be true when estimating the activity of that process for the human eye.*”

Response 15: The reviewer is totally right here, and we emphasise this issue as another limitation – and also highlighting that this study is a starting point and if more biochemical/ physiological data are available for RPE and retinal cell types, these numbers can be corrected. We plan for the future to have the data available in a database, where they are continuously updated and where other outer retina cell types will be considered.

Comment 16: “*(6) Line 453: Do the authors mean “OR” or “IS” instead of “OS”?*”

Response 16: Yes, we have corrected it to OR.

Comment 17: “*(7) Line 505: The last word in the sentence should be “anabolism”*”

Response 17: We have corrected this.

Comment 18: “(8) Line 517: No year on this citation”

Response 18: We have corrected this.

Comment 19: “*(9) There is no propagation of experimental error from original sources of data. In an ideal world, estimates of ATP expenditure by each cellular process would have error bars representing uncertainty that was propagated from the original papers. I realize this last point is perhaps too much to ask so this modification is an optional request that would enhance the manuscript but will not be required from me for publication.*”

Response 19: We have decided not to display errors bars in the visualization of ATP consumption diagrams as the ATP fluxes are not the result of one experimental uncertainty. The error associated with the ATP fluxes will be a combination of errors associated with experimental measurements (that have a quantifiable uncertainty) and assumptions that we take (that do not have a quantifiable uncertainty). [Note, that our ATP consumption rates are represented similar as previous energy budgets on the brain (PMID: 11598490) and in rod and cone PR (PMID: 19084410; PMID: 32719136) that also do not include error bars.]

However, we agree with the reviewer that these (different types of) uncertainties should be communicated to the readers. Therefore, we added a chapter at the end of the method section to explain (and quantify, where possible) the sources of uncertainties and errors. Particularly, we highlighted processes that include many assumptions that we took and that hence might be associated with larger uncertainties.

Comment 20: “*(10) To an extent the authors change the Kcat for different circadian periods. These changes often appear to be arbitrary and values are not always linked to a citation. All Kcat values should either be cited in text, or a citation should be linked to the supplemental spreadsheets. As the study stands some of the values seem to be cited but not all of them.*”

Response 20: We have double-checked to cite all kcat values exactly when mentioned in the respective main or supplemental texts.

Comment 21: “ *(11) Improvements in technology means that better retina, photoreceptor, or RPE-specific estimates of turnovers, PTMs, cytoskeletal movements, etc. might be published in the future. This study provides a framework for estimating energy expenditures that should ideally be updated as improved values for each process are determined experimentally. It is certainly not a requirement for the manuscript and is far outside my purview as a reviewer, but I highly recommend the authors consider generating a publicly available resource that uses the framework behind this study but can be updated. A web-based tool where people can deposit recommended values for a given process might be best. Such a strategy could drastically improve the usefulness and long-term accuracy of the current study.*”

Response 21: Yes, we are currently planning exactly this!

Supplementary document:
Comment 22: “ *• There are several instances in this section where assumptions are stated but the reasons for a given assumption are unstated. This leads to the reader (me) having the uneasy feeling that a substantial proportion of the calculations are determined using arbitrary starting values. In some cases the choice of constants could inform major outputs in this paper. Where possible, could the authors improve their explanations for a given assumption? This issue was noticeable in the following sections of the supplementary information: “We also assumed a higher k_cat_ (average of 0.5 and 80 s ^-1^ = 40.25 s^-1^ )”, “We initially assumed that 50 % of the proteins are degraded via the proteasome and 50 % via the lysosome”, “*”

Response 22: The reviewer is right. In some cases where no experimental information is available (particularly in the context of cytoskeletal reorganisation), we need to take assumptions – that however are consistent with other assumptions we take regarding the timing and activity of cellular processes. We made it clearer now when those assumptions are used and if possible, we extended explanations to assumptions taken.

Regarding the comment “*We initially assumed that 50% of the proteins are degraded via the proteasome and 50% via the lysosome”,*” , this is actually a nice case where we later showed that the initial assumptions where correct. The ATP flux needed for the degradation of 50 % of the proteins that need to be replaced by “demand” lead to very similar fluxes when ATP fluxes are calculated based on proteasome abundance and k_cat_! (Sometimes intuition works!)

Comment 23: “ *•* *The reference section has been added twice to this document*”

Response 23: We have corrected this.

Comment 24: “ *• For some information (for example the energetics costs from using Ca2+ ATPases), there is a range of possible ATP consumption values based on estimates and on published data. Yet in figures a single value is chosen to represent that energetic cost. Would it be feasible to represent ATP costs as a range of possible costs, where applicable? And not just for this section but wherever there is a known range of uncertain values in the calculation?*”

Response 24: All figures represent the final estimates from our work, which in some cases could be averages of our estimates and published data. This is clearly detailed in the first three datasheets of Supplementary Table S4.

Comment 25: “ *• The calculation of energetic costs associated with PTMs appear to be particularly unrealistic for a few reasons: (1) they assume every site on a protein that can be modified with a PTM is normally modified. It is normal in literature to see changes in the abundance of various PTM under different conditions, which indicates that not all possible PTM sites are normally occupied. This study would be improved by the incorporation of a term which quantifies the proportion of PTM sites that are normally occupied. (2) I think the ATP-equivalent values for production of molecules like acetyl-CoA are calculated incorrectly. The values used actually correspond with the energy that would be produced if acetyl-CoA were consumed. Use of as a PTM does not use up the energy it does not generate. The calculation should instead have the cost of Acetyl-CoA synthesis. Acetyl-CoA synthesis shares most if its reactions with glycolysis, but should additionally reduce a molecule of NAD+ to NADH. This of course assumes there is no limitation on CoA availability. When the production of a molecule used to modify a protein is energetically productive it should be subtracted from the energetic budget rather than added to it. (3) Acetylation, glycosylation, phosphorylation, and ubiquitination are far from the only posttranslational modifications. If the authors plan to only determine the energy budget associated with only these modifications, they should provide a reasonable explanation for why the other modifications are not taken into account. Perhaps other modifications are far less abundant on proteins? If so, please find and cite this information.*”

Response 25: Regarding point (1): These are the post-translational modifications for the lifetime of a protein. Hence, there will be even multiple rounds of e.g. phosphorylation and de-phosphorylation during the lifetime of the protein, which would even increase the number beyond assuming that every phosphorylation site in a protein is phosphorylated. It’s not possible to predict the dynamics of phosphorylation/dephosphorylation. In any case, PTPs have a relatively small contribution. But we emphasize this issue/limitation now in the main text.

Regarding point (2): What the reviewer is suggesting would actually result in similar ATP equivalents. We have assumed 12 ATP equivalents for Acetyl-CoA (See Table S3). The reviewer is suggesting to produce Acetyl-CoA from CoA and glycolysis. This would be pyruvate. The cost of losing a pyruvate (15 ATP) that is not available to produce energy would be compensated by 1 NADH that we are generating (3 ATP) (Table S3). So it would be exactly the same.

Regarding point (3): Yes, in fact phosphorylation, ubiquitination, glycosylation, and acetylation are the most frequent PTMs (https://awi.cuhk.edu.cn/dbPTM/statistics.php).

Comment 26: “ *• Page 19: Estimates of protein turnover may be slightly off. This paper (PMID: 7452249) is an estimate of protein synthesis for the whole rabbit retina, but average protein synthesis rates in photoreceptors are expected to be faster than for other retinal cells. If I’ve calculated correctly from this paper, the estimate for the average retina protein half-life is almost exactly 4 days. Please consider using retina-specific estimates of turnover where possible.*”

Response 26: We have used protein turnover data from retina for rPR and cPR cells, which are almost exactly what the reviewer is suggesting in his reference (4 days = REVIEWER vs 5.4 days = AUTHORS). For RPE, we used estimates from liver.

Comment 27: “ *• Page 21: I think “24 night and dark cycles” is meant to mean “24-hour light and dark cycle”*”

Response 27: We have corrected this.

Reviewer #3: Strengths.

“ *• This paper presents a comprehensive list of likely energy demands on photoreceptors and RPE cells. Then accompanying paper presents a corresponding list of the ways that fuels can be supplied to these cells and how the energy can be extracted from them and stored in ATP. The authors obviously have put a huge effort into bringing all this information together in these lists.*
*• The comprehensive nature of this study can be a starting point for quantitative modeling of energy supply and demand in the retina and RPE. Most of the data used are not really relevant for these ocular tissues, but more relevant data can be updated into this model as the authors and others recognize the usefulness of data from direct physiological and biochemical measurements that have been made and will become available on human and mouse ocular tissues and tissues from other species.*”

Weakness.
Comment 28: “ *• The data used for these models in many cases are not appropriate or relevant. For example, it cannot be accurate to predict enzymatic activities from transcript levels. Translation of the transcripts is different for each protein, the stability of each enzyme is different and the enzymes are regulated by posttranslational modifications. For example light activated rhodopsin is inactivated by phosphorylation and there is so much rhodopsin in a rod that substantial amounts of ATP can be consumed in this process. That is an example of an energy demand that is unique to photoreceptors. The locations of enzymes in photoreceptors can change in light and darkness. These factors are relevant but not sufficiently considered in this model.*”

Response 28: We agree with the reviewer that estimating protein abundances from mRNA abundances has limitations. However, as there is a high dynamic range both for mRNA and protein abundances, correlations between the two are generally in the range of <0.7**–**0.8 (REF Aebersold, Cell). Hence, generally, the order of magnitude will be correct. We have seen this is case of ribosomes, where the number of ribosomal particles calculated from protein expression (estimated from mRNA) agrees well with the number of ribosomal particles calculated based on the average weight of rRNA in cells. Nevertheless, the reviewer is right that estimating protein levels from mRNA has its limitations, as there might be individual proteins that have significantly higher or lower protein abundances as estimated from mRNA. However, in the absence of quantitative single-cell proteomic data, there is no real solution to the problem.

We have added now a paragraph in the discussion to emphasize limitations arising from this problem.

With respect to the comment about ATP requirement for the phosphorylation of rhodopsin, phosphorylation of light-activated rhodopsin was already taken into account in our energy budget (See in supplement “Step 4 – Phosphorylation of Rhodopsin by Rhodopsin kinase”). We show that under indoor light conditions, and assuming 3 phosphorylations per active rhodopsin, the energy costs are very small (1.8 x 10^3^ ATP s^-1^ per rod). See also the detailed response in comment 30.

Comment 29: “*“Overall, this is a useful starting point for this type of analysis and I recommend that the authors emphasize the purpose of this analysis in this way – that future analyses will incorporate actual data from biochemical and physiological studies of retina and RPE tissues into their model.*”

Response 29: We thank the reviewer for this. Indeed, this works represents a starting point and – since all data, assumptions, and detailed calculations are available in the supplementary materials – if novel biochemical data from biochemical and physiological studies of retina/RPE are available, the energy budget can be re-calculated in a straightforward way.

We have added a paragraph to the discussion that emphasises this.

“*The authors should address a few specific comments and suggestions.*”

Comment 30: “*1. Lines 303-306: Rhodopsin phosophorylation is a major consumer of ATP. It only happens in light but there may be continuous phosphorylation and dephosphorylation when light is being absorbed.*”

Response 30: We have already taken rhodopsin phosphorylation into consideration, and it is not a great consumer of ATP at indoor conditions (1.8 x 10^3^ ATP s^-1^ per rod) (see supplement). We have assumed 3 phosphorylation sites per activated rhodopsin, which is close to what is proposed in Hurley et al, 1998 (PMID: 966700) (“40% of rhodopsin typically remains unphosphorylated” and “8–9 phosphates have been detected on a rhodopsin molecule”). We assumed that once a rhodopsin molecule is activated, it is phosphorylated and then dephosphorylated. However, even if there would be continues phosphorylation and deactivation on an individual rhodopsin, it would not increase the budget very much as dephosphorylation is in the range of 30 min to 1 h (PMID: 7782279).

Comment 31: “*2. In the discussion section please make a direct comparison of the current findings and conclusions with the estimates of ATP consumption that were reported by Okawa et al in 2008.*”

Response 31: We have done that.

Comment 32: “*3. lines 347-353. It would be helpful to discuss the importance of mitochondrial coupling. Photoreceptor mitochondria may be more uncoupled than in most other types of cells. The authors cite reference 46, which is based on measurements of mitochondria from cultured cells and from mitoplast preparation. The properties of mitochondria can vary in the degree to which they are uncoupled and photoreceptor mitochondria may be particularly unique in this way. This should be discussed.*”

Response 32: We discuss this now at the point mentioned by the reviewer. We also discuss this now in the context of energy supply in the accompanying paper.

Comment 33: “*4. line 378 - please define what PDE delta is.*”

Response 33: We have defined this enzyme now.

Comment 34: “*5. line 42- delete "on"*”

Response 34: There is no “on” in line 42. Maybe the reviewer was referring to an “of” that had to be deleted in line 34 (abstract)?
